# Déjà Vu in *Merodon* Taxonomy (Diptera: Syrphidae): Unveiling Hidden Species Inside *Merodon caudatus* and *M. ottomanus* Taxa †

**DOI:** 10.3390/insects16101009

**Published:** 2025-09-28

**Authors:** Ante Vujić, Laura Likov, Nataša Kočiš Tubić, Mihajla Djan, Antonio Ricarte, Santos Rojo, Celeste Pérez-Bañón, Jelena Ačanski, Andrijana Andrić, Tamara Tot, Snežana Radenković

**Affiliations:** 1 Department of Biology and Ecology, University of Novi Sad, Trg Dositeja Obradovića 2, 21000 Novi Sad, Serbia; ante.vujic@dbe.uns.ac.rs (A.V.); natasa.kocis@dbe.uns.ac.rs (N.K.T.); mihajla.djan@dbe.uns.ac.rs (M.D.); tamarat@dbe.uns.ac.rs (T.T.); snezana.radenkovic@dbe.uns.ac.rs (S.R.); 2 Research Institute CIBIO (Centro Iberoamericano de la Biodiversidad), Science Park, University of Alicante, Ctra. San Vicente del Raspeig s/n, 03690 Alicante, Spain; antonio.ricarte@ua.es (A.R.); 3 Department of Environmental Sciences & Natural Resources, University of Alicante, 03690 Alicante, Spain; santos.rojo@ua.es (S.R.); celeste.perez@ua.es (C.P.-B.); 4 BioSense Institute, University of Novi Sad, Dr Zorana Ðinđića 1, 21000 Novi Sad, Serbia; acanski@biosense.rs (J.A.); andrijana.andric@biosense.rs (A.A.)

**Keywords:** pollinators, hoverflies, taxonomic revision, new species, species groups, taxonomy

## Abstract

Two new species groups within the *avidus-nigritarsis* lineage of the hoverfly genus *Merodon* Meigen are here defined and assessed, i.e., the *Merodon caudatus* and *Merodon ottomanus* groups. Within the *M. caudatus* group, two species are recognised:*Merodon caudatus* Sack and a new species, *Merodon crispotarsus* sp. nov. Within the *M. ottomanus* group, an extensive examination of material from different collections revealed the presence of four new species, *Merodon auriolus* sp. nov., *Merodon paeninsula* sp. nov., *Merodon projectus* sp. nov., and *Merodon rostrum* sp. nov., apart from *Merodon ottomanus* Hurkmans. The genetic characterisation of species from the *M. ottomanus* group was performed through Maximum Parsimony (MP) and Maximum Likelihood (ML) analyses of the 5′ end of mitochondrial COI gene sequences, and the existence of the five above-mentioned independent taxa was supported. In addition, we confirmed the validity of *M. auriolus* sp. nov., *M. paeninsula* sp. nov., and *M. ottomanus* by using an integrative taxonomic approach including wing shape differences. Diagnoses, keys for both species groups, and the species they consist of, as well as distribution maps for all studied species, are provided.

## Simple Summary

The remarkable diversity and species richness of the *Merodon avidus–nigritarsis* lineage has been further expanded with the discovery of five new species, which were distinguished using an integrative approach including traditional adult morphological characteristics, molecular analysis, and geometric morphometrics.

## 1. Introduction

With more than 200 species described worldwide, the genus *Merodon* Meigen (Diptera, Syrphidae, Merodontini) is one of the largest within the family [1,2,3,4,5,6,7,8,9]. It has a Palaearctic and Ethiopian range [1], but one species, *Merodon equestris* (Fabricius), has been introduced in the Nearctic Region and New Zealand, where it causes pests in ornamental plants [10]. The northern and eastern parts of the Mediterranean Basin have the highest species diversity reported in *Merodon*, probably due to their high diversity of plants (geophytes) hosting this genus’ larvae [11,12,13].

Recent studies propose the division of the genus *Merodon* into five monophyletic lineages (*albifrons*, *aureus*, *avidus-nigritarsis*, *desuturinus*, and *natans*), 24 species groups, 2 species subgroups, and 10 unplaced species [1]. For the *avidus-nigritarsis* lineage, 10 species groups have been defined (*aberrans*, *aurifer*, *avidus*, *clavipes*, *fulcratus*, *italicus*, *nigritarsis*, *pruni*, *serrulatus*, and *tarsatus*), and 8species have been found to be outliers within the lineage: *M. auronitens* Hurkmans, *M. caudatus* Sack, *M. clunipes* Sack, *M. crassifemoris* Paramonov, *M. eumerusi* Vujić, Radenković et Likov, *M. hirtus* Sack, *M. murinus* Sack, and *M. ottomanus* Hurkmans. A total of 8 out of the 10 mentioned groups have already been revised:the *aurifer* group [2], the *avidus* [8,14,15,16], the *nigritarsis* [16], the *serrulatus* [17], the *aberrans* [6], the *tarsatus* group [7], the *pruni*, and the *clavipes* species groups [9].

Of the eight outlier species of the *avidus-nigritarsis* lineage, *M. caudatus* and *M. ottomanus* are to be treated in the present paper. *Merodon caudatus* is a species with partly reddish terga and a unique metaleg morphology consisting of a metatibia twisted medially in the apical half and a strongly modified metabasotarsomere. This species has been reported inIsrael, Turkey, and Palestine [1]. *Merodon ottomanus* is a species with a dark abdomen, reddish-yellow basoflagellomere, usually yellow metatarsi (at least basotarsomere), large and rounded posterior surstylar lobe, and small anterior surstylar lobe. The distribution of *M. ottomanus* is fragmented and includes the Iberian Peninsula, Peloponnesus (Greece), Turkey, and Iran [1].

The resolution of the taxonomic status for the majority of groups mentioned previously has been achieved through integrative taxonomy. In hoverfly taxonomy, this approach mainly combines the results of morphological, molecular, and morphometric analyses, sometimes also with data on ecological preferences and distribution patterns. The COI barcode and other molecular characters such as Cyt b, 28S rRNA gene, and ITS region have been frequently used for molecular assessment [5,18,19,20,21,22]. For geometric morphometric studies, traits such as the shape of the wing, male genitalia, and spiracular openings in larvae have proven particularly useful [4,15,19,20,23,24,25,26,27,28]. These characters have been instrumental in the taxonomy of the genus *Merodon* and other hoverflies, as well as in various other insect groups. Consequently, integrative taxonomy has been used once again to support our morphological findings and to explore the taxonomic variability within the species recognised so far as *M. ottomanus* and *M. caudatus*.

After a detailed examination of the material available forboth *M. caudatus* and *M. ottomanus*, the authors of the present paper found that these two species were, in fact, groups of species and thus, two new species groups could be defined within the *avidus-nigritarsis* lineage. Therefore, the aims of the present study were as follows: (1) to revise the specimens labelled as *M. caudatus* and *M. ottomanus* in different entomological collections; (2) to define the *caudatus* and *ottomanus* species groups and fix the list of species they consist of; (3) to illustrate the distribution of the examined material of all species; and (4) to build identification keys to species for both novel groups.

## 2. Materials and Methods

### 2.1. Taxonomy

The studied material is deposited in the following museums and entomological collections: BM coll.—Barták Miroslav collection, Prague, Czech Republic; BMNH—Natural History Museum, London, UK; CEUA-CIBIO—Colección Entomológica de la Universidad de Alicante, Alicante, Spain; DD coll.—Dieter Doczkal collection, Munich, Germany; EMIT—Entomological Museum of Isparta, Isparta, Turkey; FSUNS—Faculty of Sciences, Department of Biology and Ecology, University of Novi Sad, Novi Sad, Serbia; MMH coll.—Maleki Milani Hasan collection, Tabriz, Iran; NBCN—Naturalis Biodiversity Center, Leiden, The Netherlands; SIZK—I. I. Schmalhausen Institute of Zoology of the National Academy of Sciences of Ukraine, Kyiv, Ukraine; STJ coll.—Smit T. John collection, The Netherlands; TAU—Tel Aviv University, Tel Aviv, Israel; ZFMK—Zoologisches Forschungsinstitut und Museum Alexander Koenig, Bonn, Germany.

Dry specimens were relaxed in a humidity chamber in order to study the male genitalia. Once relaxed, terminalia were extracted with a hook-tipped entomological pin. After that, 5 min boiling in water-diluted KOH pellets followed, and then genitalia were briefly immersed in acetic acid to neutralise the KOH excess, and finally in ethanol to remove the acid excess.

The morphological terminology follows Thompson [29], except for the male genitalia, which follows Marcos-García, Vujić & Mengual [30]. ‘Surstylar lobe’ is used instead of ‘surstyle lobe’. In the lab, photographs of specimens or parts of them were taken with a Nikon Digital Sight 10 digital camera attached to a Nikon SMZ18 stereomicroscope. Individual photos of the same element were stacked using CombineZ software version 5 [31]. For drawings, a Leica MZ16 binocular microscope was used with an FSA 25 PE drawing tube. Distribution maps were generated with the mapping software QGIS 3.30.0 (QGIS.org).

### 2.2. Geometric Morphometrics

Wing shape variation was studied in 29 specimens of the *M. ottomanus* group from Greece, Spain, and Turkey. The right wing of each specimen was dissected using microscissors under a Nikon SMZ18 stereomicroscope and mounted on a microscopic slide using Hoyer’s medium. Wings were photographed using a Nikon DS-Fi3 camera attached to a Nikon SMZ18 stereomicroscope and labelled and archived with a unique code in the FSUNS database.

**Figure 1:**
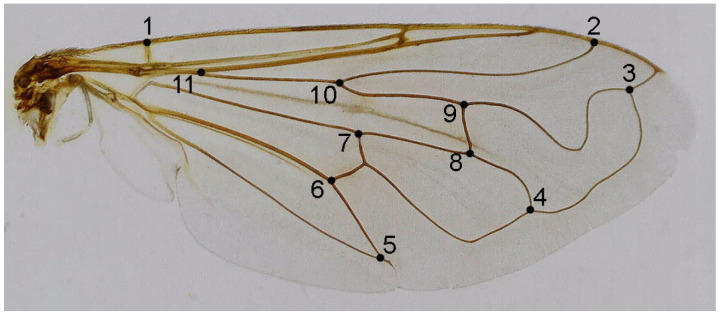
*Merodon ottomanus*, the location of 11 landmarks on the right wing selected for geometric morphometric analysis.

Eleven homologous landmarks, evenly distributed across the wing, were digitised using TpsDig ver. 2.31 [32] (Figure 1). Generalised least squares (GLS) Procrustes superimposition on the raw coordinates was performed using TpsRelw ver. 1.68 [33] to minimise non-shape variations in the location, scale, and orientation of wings and to superimpose the wings in a common coordinate system [34,35]. Principal component analysis was carried out on the Procrustes shape variables to reduce the dimensionality of the data set. All further statistical analyses were conducted in the reduced space using a subset of independent principal components (PCs) that describe the highest overall classification percentage calculated in stepwise discriminant analysis [36]. To explore wing shape variation among the taxa, we employed canonical variate (CVA) and discriminant function analysis (DFA). Phenetic relationships among taxa were determined by UPGMA analysis based on squared Mahalanobis distances computed from the discriminant function analysis applied to wing shape variables. All statistical analyses were performed in Statistica for Windows [37].

### 2.3. Molecular Analysis

The specimens analysed molecularly are included in Table S1. Genomic DNA was extracted from two or three legs of each specimen using a slightly modified SDS extraction protocol [38]. The 5′-end of the mitochondrial protein-coding cytochrome c oxidase subunit I (COI) gene was amplified. For this purpose, we used the LCO1490 and HCO2198 primer pair [39]. The PCR reaction was carried out according to Kočiš Tubić et al. [19]. The amplification products were enzymatically purified by Exonuclease I and FastAP Thermosensitive Alkaline Phosphatase enzymes (ThermoScientific, Vilnius, Lithuania) and sequenced in the forward direction by the Macrogen*ez-Seq* service (Macrogen Europe, Amsterdam, The Netherlands).

The 5′-COI gene sequences obtained were edited for base-call errors using BioEdit v. 7.2.5. [40] and adjusted manually. Additional sequences of *Eumerus amoenus* Loew and *Merodon desuturinus* Vujić, Šimić et Radenković served as outgroups, were retrieved from GenBank and joined to the sequence matrix (see Table S1 for GB accession numbers of all analysed ingroup and outgroup species). Alignment of the sequences was achieved using the Clustal W algorithm [41] implemented in BioEdit [40]. All sequences in the analysed data set were trimmed to equal length. The Maximum Parsimony (MP) tree was constructed using NONA [42], spawned with the aid of ASADO, version 1.85 [43], using the heuristic search algorithm (settings: multx1000, hold/100, max trees 100,000, TBR branch swapping). Nodal support was estimated using nonparametric bootstrapping with 1000 replicates. The tree was rooted in *Eumerus amoenus*.

## 3. Results

### 3.1. Taxonomic Revision

#### 3.1.1. *Merodon avidus-nigritarsis* Lineage

**Figure 2:**
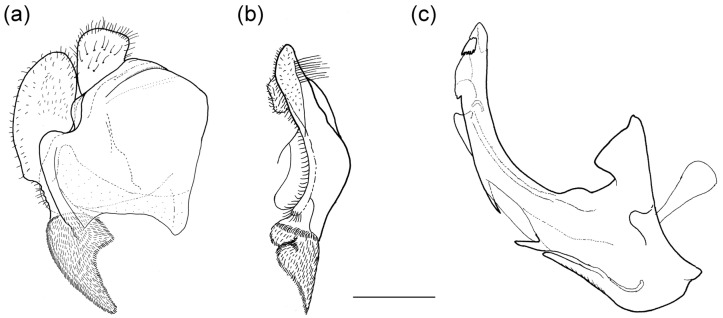
Male genitalia of *Merodon nigritarsis*. (**a**) lateral view of epandrium; (**b**) ventral view of epandrium; (**c**) lateral view of hypandrium. Scale bar: 0.5 mm.

**Diagnosis**. Medium to large-sized species (11–20 mm), usually with white pollinose vittae on scutum and white-pollinose fasciate maculae on terga; anterior anepisternum bare below postpronotum; abdomen elongate, usually narrow and tapering, longer than scutum and scutellum together; posterior part of mesocoxa usually without long pile; basoflagellomere usually, at most, twice as long as wide; male genitalia: anterior surstylar lobe usually rhomboid-shaped, covered with dense short pile; posterior surstylar lobe usually longer than anterior one; interior accessory lobe of posterior surstylar lobe narrow and long; cercus rectangular, without prominences; hypandrium usually narrow, elongate and sickle-shaped; posterior end of lateral sclerite of the aedeagus tapering; theca of hypandrium usually with a pair of lateral projections; lingula developed (Figure 2).

This lineage includes 10 species groups plus the newly defined *M. caudatus* and *M. ottomanus* groups.

#### 3.1.2. *Merodon caudatus* Group

**Figure 3:**
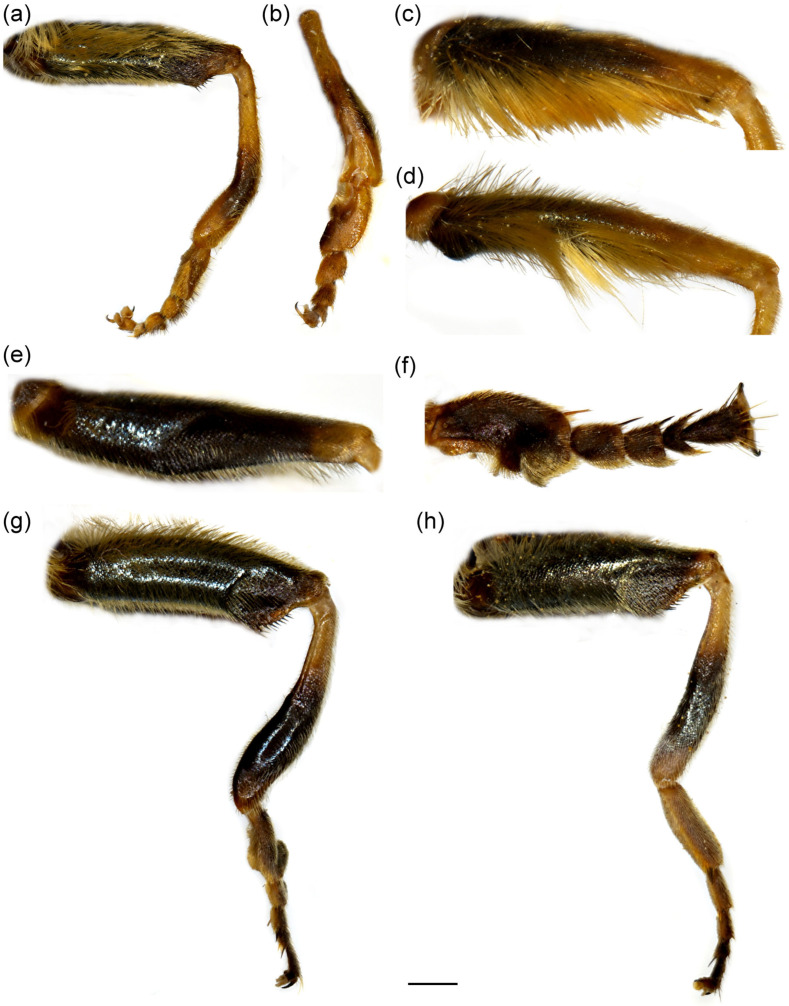
Parts of legs. (**a**) *Merodon caudatus*, metaleg of male; (**b**) *M. caudatus*, metatibia and metatarsus of male; (**c**) *M. caudatus*, profemur of male; (**d**) *M. caudatus*, mesofemur of male; (**e**) *M. crispotarsus* sp. nov., mesofemur of male; (**f**) *M. crispotarsus* sp. nov., basitarsus of male; (**g**) *M. crispotarsus* sp. nov., metaleg of male; (**h**) *M. crispotarsus* sp. nov., metaleg of female. (**a**–**e**,**g**,**h**) lateral view; (**f**) dorsal view. Scale bar (**a**,**g**,**h**) 1 mm; (**b**,**f**) 0.5 mm; (**c**–**e**) 0.8 mm.

**Figure 4:**
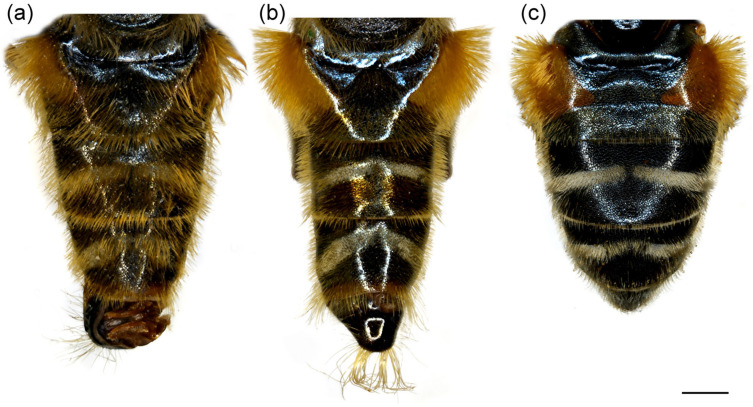
Dorsal view of abdomen. (**a**) *Merodon caudatus*, male; (**b**) *M. crispotarsus* sp. nov., male; (**c**) *M. crispotarsus* sp. nov., female. Scale bar: 1 mm.

**Figure 5:**
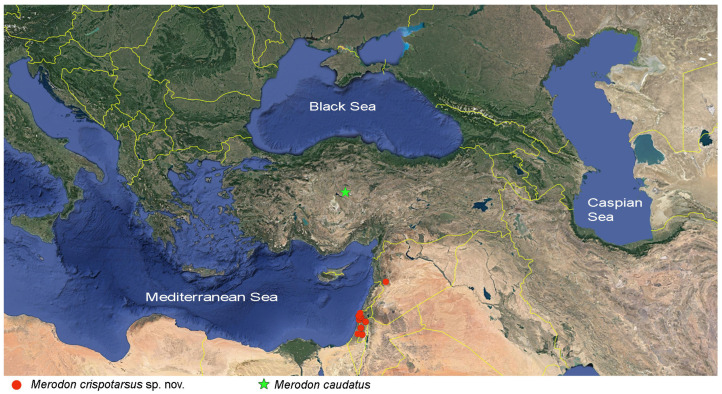
Distribution map of the *Merodon caudatus* species group.

**Diagnosis**. Species with a unique metaleg morphology among the species of the *avidus-nigritarsis* lineage: metatibia twisted in the apical half (as in Figure 3a), metabasotarsomere strongly modified in males (as in Figure 3b), less in females (as in Figure 3h); tergum 2 with a pair of reddish yellow lateral, triangular maculae (as in Figure 4a). Species of this group are restricted to Turkey, Israel, Syria, and Palestine (Figure 5).

***Merodon caudatus*** **Sack, 1913**

**Figure 6:**
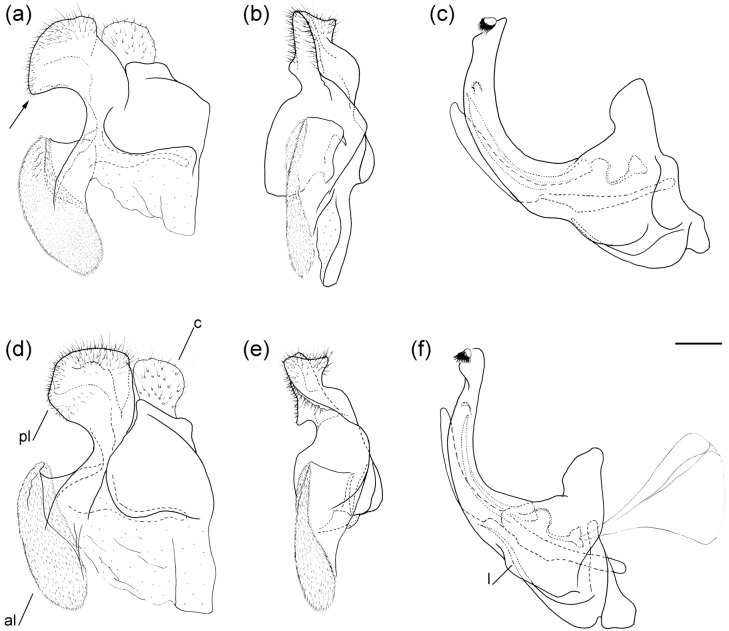
Male genitalia of (**a**–**c**) *Merodon caudatus*; (**d**–**f**) *M. crispotarsus* sp. nov. (**a**,**d**) lateral view of epandrium; (**b**,**e**) ventral view of epandrium; (**c**,**f**) lateral view of hypandrium. Abbreviations: al—anterior surstyle lobe; c—cercus; l—lingula; pl—posterior surstyle lobe. The hammer-like posterior surstylar lobe is marked with an arrow. Scale bar 0.5 mm.

**Diagnosis**. Pro- and mesotrochanters yellowish red; metatibia curved (swollen) in apical third, enlarged at tip (Figure 3a); all tarsi yellow, covered dorsally with erect pilosity (Figure 3a); male pro- and mesofemora (Figure 3c,d) posteriorly, and metafemur anteriorly with dense, strong, yellow pilosity; mesofemur with basal bulge ventrally (Figure 3d); all tarsomeres of metatarsus strongly modified (Figure 3b); male genitalia: in Figure 6a–c: hammer-like posterior surstylar lobe (Figure 6a: marked with an arrow).

**Material examined**. **Neotype**: 1♂, 01.vi.1972, leg. M. Doğanlar, [‘Type of *M. claudius*; MUS; 2♂♂/? aureotibia/Hurkm.’], FSUNS.

**Note.** The holotype from ‘Asia Minor’ (Turkey) was considered lost by Hurkmans [44], and it was not located in the frame of the present study. Thus, we here designate a neotype based on material from the type locality (Turkey).

**Additional material**: TURKEY: 1♂, 01.vi.1972, leg. M. Doğanlar, [‘Type of *M. claudius*; MUS’], FSUNS (FSUNS ID 02226, ‘AM-05-58’); 1♀, 01.vi.1972, leg. M. Doğanlar, [‘MUS’], FSUNS (FSUNS ID 02227, ‘AM-05-227’).

**Distribution.** Turkey (Figure 5).

**

***Merodon crispotarsus* Vujić, Likov et Radenković sp. nov.**

urn:lsid:zoobank.org:act:2FACA83F-FAD6-4147-AD70-9D5DF7039393

**Material examined**. **Holotype**: 1♂, ISRAEL, Gilboa, 01.iv.1998, leg. A. Freidberg, FSUNS (FSUNS ID 04901).

**Paratypes**: ISRAEL: 1♂, Mount Carmel, Bat Shlomo, 32.5967° N 35.0019° E, 22.iii.1981, leg. N.B.M. Brantjes, [det. as *Merodon caudatus* Sack, 1913 by A. Vujić (2008) and by J.A.W. Lucas], NBCN (FSUNS ID 02224, ‘AM-05-23’); 4♂♂, 1♀, Tivon, 03.iv.1973, leg. F. Nachbar, TAU; 4♂♂, 1♀, Tivon, 02.iv.1975, leg. F. Kaplan, TAU; 1♀, Tivon, 02.iv.1975, leg. F. Kaplan, [det. as *Merodon caudatus* Sack, 1913 by W. Hurkmans (1994) and by A. Vujić], TAU (FSUNS ID 04958); 1♂, same data as for preceding, TAU (FSUNS ID 04916); 1♀, Mount Carmel [Har Karmel], M. Haifa, 32.7286° N 35.0467° E, 16.iv.1980, leg. I.A.W. Lucas, [det. as *Merodon caudatus* Sack, 1913 by W. Hurkmans (1995) and by A. Vujić (2008)], NBCN (FSUNS ID 02225, ‘AM-05-24’); 1♂, Carmel, Haifa, 10.iv.1947, TAU; 1♂, Haifa, 26.iii.1977, leg. A. Freidberg, [det. as *Merodon caudatus* Sack, 1913 by W. Hurkmans (1994) and by A. Vujić], TAU (FSUNS ID 04963); 1♂, Haifa, 12.iv.1963, leg. Kugler, [“Kugler *L. caudata*”], TAU; 1♀, Gilboa, 17.iii.1978, leg. Kugler, TAU; 6♂♂, 3♀♀, Gilboa, 01.iv.1998, leg. A. Freidberg, TAU; 1♂, Gilboa, 01.iv.1998; leg. A. Freidberg, ‘*M. caudatus*’, TAU (FSUNS ID 04888); 1♀, same data as for preceding, FSUNS (FSUNS ID 04889); 1♀, Gilboa, 01.iv.1998, leg. A. Freidberg, FSUNS (FSUNS ID 04967); 1♀, Gilboa, 01.iv.1998, leg. A. Freidberg, [det. as *Merodon caudatus* Sack, 1913 by A. Vujić], TAU (FSUNS ID 04964); 1♀, Jerusalem, KiriethSemhuel, 05.iv.1936, TAU; 1♂, Jerusalim, Wadi RuazBethakerem, 24.iii.1951, leg. O. Theodor, TAU; 1♂, same data as for preceding, 20.iv.1953, TAU; 1♀, Bat Jamal, 23.iii.1968, TAU; 1♂, Ga’aton, 33.0062° N 35.2145° E, 21.iv.1973, leg. M. Kaplan, TAU; 1♀, same data as for preceding, [“M. Kaplan *M. caudatus*”], TAU; 1♂, W. Faria, 01.iii.1973, leg. A. Freidberg, TAU; 2♂♂, same data as for preceding, leg. M. Kaplan, TAU; 1♂, same data as for preceding, [“M. Kaplan *M. caudatus*”], TAU; 1♂, W. Ara, 23.iii.1974, leg. F. Kaplan, TAU; 2♀♀, same data as for preceding, leg. F. Nachbar, TAU; 1♂, Nahshonim, 20.iii.1974, leg. F. Kaplan, [“Sack, *M. caudatus*”], TAU; 2♂♂, 2♀♀, Gollani Jun., 23.iii.1974, leg. M. Kaplan, TAU; 1♂, same data as for preceding, 28.iii.1974, TAU; 1♂, Zova, 31.iii.1974, leg. M. Kaplan, TAU; 1♀, Ma’ale Hachamicha, 30.iii.1974, leg. F. Nachbar, TAU; 1♂, Beit Guvrin, 27.iii.1976, leg. A. Freidberg, TAU; 1♂, 1♀, Zecharia, 26.iii.1978, leg. A. Freidberg, TAU; 1♂, Yarbi, 21.ii.1978, leg. M. Kaplan, [“found fresh pupa few days later stigmae appeared”], TAU; 1♀, Har Sumaq (Carmel), 25.iii.1979, leg. Kugler, TAU; 1♂, Ma’agan Michael dunes, 28.iii.1984, [“C. O’Toole Aiincopvith B. museum visit 09/2010”], TAU; 1♂, Carmel, 25.iii.1984, leg. A. Hefetz, TAU; 1♀, Galin w., 23.iii.1986, leg. Sheny—dor E., TAU; 1♀, Zikron Ya’acov, 01.iv.1998, leg. A. Freidberg, TAU; 3♂♂, Zomet Haela, 02.iv.1999, leg. A. Freidberg, TAU; 1♀, Shoham, 18.iii.2005, leg. C. Grach, TAU; 1♀, Shoham, 23.iii.2007, leg. K. Levi, T. Hughes Games, TAU; 1♂, Shoham, 04.iv.2009, leg. G. Wizen, TAU; 1♂, Jerusalem, Beit HaKerem, 31.7833° N 35.2° E, 17.iii.1951, leg. O. Theodor, [det. as *Lampetia caudata* Sack by V. Doesburg, det. as *Merodon caudatus* Sack, 1913 by A. Vujić 2008], [‘Museum Leiden/Collectie Van Doesburg/rec. 1973’], NBCN (FSUNS ID 04087); 1♀, same data as for preceding, NBCN (FSUNS ID 04088); 1♂, Jerusalem, Beit HaKerem, 17.iii.1951, leg. O. Theodor, TAU; 1♂, 1♀, same data as for preceding, [det. as *Lampetia caudata* by Doesburg, det. as *Merodon caudatus* by Hurkmans Willem], TAU; 1♂, 1♀, Copula Beit HaKerem Jerusalem, 07.v.1949, leg. O. Theodor, TAU; 1♂, Chanita, 08.iv.1946, leg. Bylinski Salz, TAU.

STATE OF PALESTINE: 1♀, Yakir, 04.iv.1981, leg. A. Freidberg, FSUNS (FSUNS ID 04975); 2♀♀, same data as for preceding, TAU; 1♀, Benjamina, 16.iii., leg. Bylinski Salz, TAU; 1♂, Elon, 21.iii.1946, leg. Bylinski Salz, TAU; 1♂, Shuneh Bentaminah coastal plane, 08.iv.1953, leg. Theodor O., TAU; 1♂, 1♀, Shune coastal plane, 07.iv.1954, leg. Theodor O., TAU; 1♀, same data as for preceding, 08.iv.1954, TAU.

**Additional material**: ISRAEL: 1♂, Jerusalem, Beit HaKerem, 31.7833° N 35.2° E, 17.iii.1951, leg. O. Theodor, [det. as *Lampetia caudata* by P.H. van Doesburg, ‘P.H. van Doesburg collection rec. 1973’, det. as *Merodon caudatus* by A. Vujić], NBCN; 1♀, same data as for preceding, NBCN; 1♂, 1♀, Mount Carmel, Bat Shlomo, 32.5967° N, 35.0019° E, 22.iii.1981, NBCN; 1♂, same data as for preceding, [“Neotype of *Merodon caudatus* by Hurkmans 1988”, “J.A.W. Lucas collection”], NBCN.

SYRIA: 1♀, Orontes, 30 km SW Homs, 500 m, 15.iv.1992, leg. Warncke, ZFMK (FSUNS ID 25345, ZFMK-DIP-00069636).

**Note.** All available material of the *Merodon caudatus* group from museums was identified as *Merodon caudatus*. We discovered the existence of an additional species under this name, and this additional species resulted in having a different distribution than *M. caudatus*. The species from Turkey is the typical *M. caudatus* based on the type locality, whilst the species from Israel, Syria, and Palestine is described here as a new species, *M. crispotarsus* sp. nov.

**Diagnosis**. Mesofemur enlarged ventro-medially (Figure 3e); metatibia twisted in the apical fourth (Figure 3g); metabasotarsomere black at least dorsally; metatarsomeres with strong black setulae dorso-laterally (Figure 3f); metabasotarsomere modified; other leg tarsomeres of the usual shape; in male tergum 5 modified, protruded with a pair of apical tufts of long yellow pile (Figure 4b).

**Description**

**Male**

**Figure 7:**
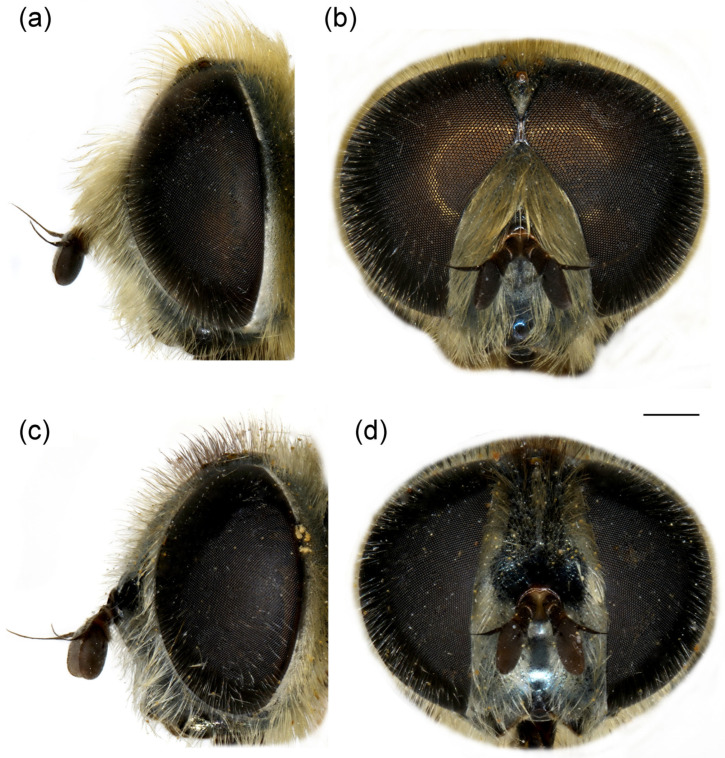
*Merodon crispotarsus* sp. nov. (**a**) head of male, dorsal view; (**b**) head of male, frontal view; (**c**) head of female, dorsal view; (**d**) head of female, frontal view. Scale bar: 1 mm.

**Head**. Antenna black; basoflagellomere elongated, about 1.5× longer than wide (Figure 7a); fossette large, dorso-lateral; arista thickened at basal third; face and frons black, with dense greyish pollinosity, face covered with dense pale yellow pilosity; pile on frons yellowish; oral margin shiny black, without pollinosity; lunula black to brown, bare; eye contiguity about 5–6 facets long; vertical triangle isosceles, shiny, black, covered with yellowish pilosity; ocellar triangle equilateral; occiput with yellowish pile, grey pollinose; eyes covered with long, whitish-grey pile.

**Figure 8:**
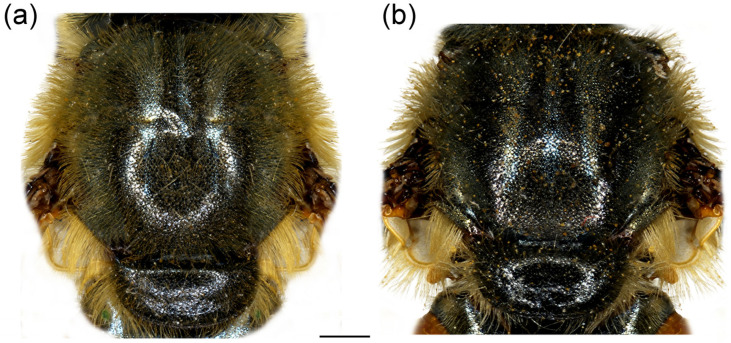
Thorax of *Merodon crispotarsus* sp. nov., dorsal view. (**a**) male; (**b**) female. Scale bar: 1 mm.

**Thorax**. Scutum and scutellum black with brownish lustre, covered with long yellow to pale yellow pile; scutum with indistinct pollinose vittae (Figure 8a); posterodorsal part of anterior anepisternum, posterior anepisternum (except anteroventral angle), anterior anepimeron, dorsomedial anepimeron, and posterodorsal and anteroventral parts of katepisternum with longer, dense whitish to yellow pile; wings mostly covered with microtrichia; wing veins brown to light brown; calypter and halter yellowish; legs black, except yellow apex of femora and basal third of tibiae; basal tarsomeres of proleg and mesoleg brown to yellow; basotarsomere of metaleg brown ventrally; apex of tibiae and tarsomeres of proleg and mesoleg with setulae, reddish on proleg and black on mesoleg; mesofemur enlarged ventro-medially (Figure 3e); metatibia twisted in apical fourth (Figure 3g); tarsomeres of metaleg with strong black setulae dorso-laterally (Figure 3f); only basotarsomere of metaleg modified, other tarsomeres of the usual shape; femora and tibiae covered with yellow to whitish pile.

**Abdomen**. Elongated, about 1.3× longer than mesonotum; terga black, except for the reddish lateral, triangular maculae on tergum 2; terga 3–4 with a pair of grey pollinose fasciae (Figure 4b); pilosity on the abdomen long, yellow to reddish, medially on terga shorter; pile very long on sternum 4 and tergum 4; tergum 5 modified, protruded with a pair of apical tufts of long yellow pile (Figure 4b).

**Male genitalia** (Figure 6d–f). Anterior surstylar lobe large, elongated, covered with short pile (Figure 6d: al); posterior surstylar lobe rounded (Figure 6d: pl) cercus oval (Figure 6d: c); hypandrium sickle-shaped, without lateral projections; lingula present (Figure 6f: l).

**Female**. Similar to male except for typical sexual dimorphism and for the following features: frons with broad pollinose vittae along eye margins, black pilose at the level of ocellar triangle (Figure 7c,d); metatibia less twisted (Figure 3h); basotarsomere of metaleg less modified (Figure 3h).

**Etymology**. The specific epithet is formed from the Latin ‘crispum’, meaning curled/curly, plus ‘tarsus’, referring to the terminal part of a leg.

**Distribution**. Western parts of the Levant region, in Israel, Syria, and Palestine (Figure 5).

**

**Key to the species of *M. caudatus* group**

1. All tarsi yellow, covered dorsally with erect pilosity (Figure 3a); in male pro- and mesofemora posteriorly, and metafemur anteriorly with dense, strong, yellow pilosity (Figure 3a,c,d); mesofemur with basal bulge ventrally (Figure 3d); all tarsomeres of metatarsus strongly modified (Figure 3b); tergum 5 with the usual shape (Figure 4a) ........................................................................................................***Merodon caudatus* Sack, 1913**
- Tarsi partly black, without erect pilosity dorsally (Figure 3g); in male, femora without dense, strong, yellow pilosity (Figure 3e,g); only basotarsomere of metaleg strongly modified (Figure 3f), other tarsomeres of the usual shape; tergum 5 modified, protruded, with a pair of apical tufts of long yellow pile (Figure 4b) .... ***Merodon crispotarsus* sp. nov.**

#### 3.1.3. *Merodon ottomanus* Group

**Figure 9:**
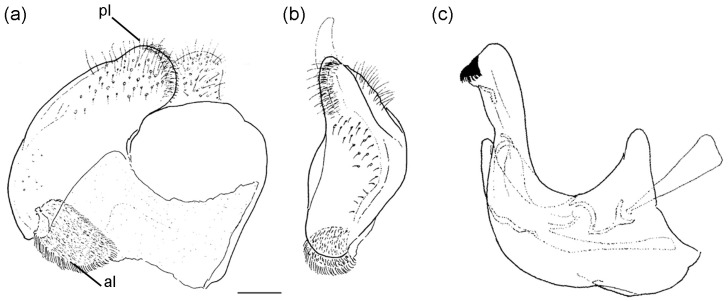
Male genitalia of *Merodon ottomanus*. (**a**) lateral view of epandrium; (**b**) ventral view of epandrium; (**c**) lateral view of hypandrium. Abbreviations: al—anterior surstylar lobe; pl—posterior surstylar lobe. Scale bar: 0.2 mm.

**Figure 10:**
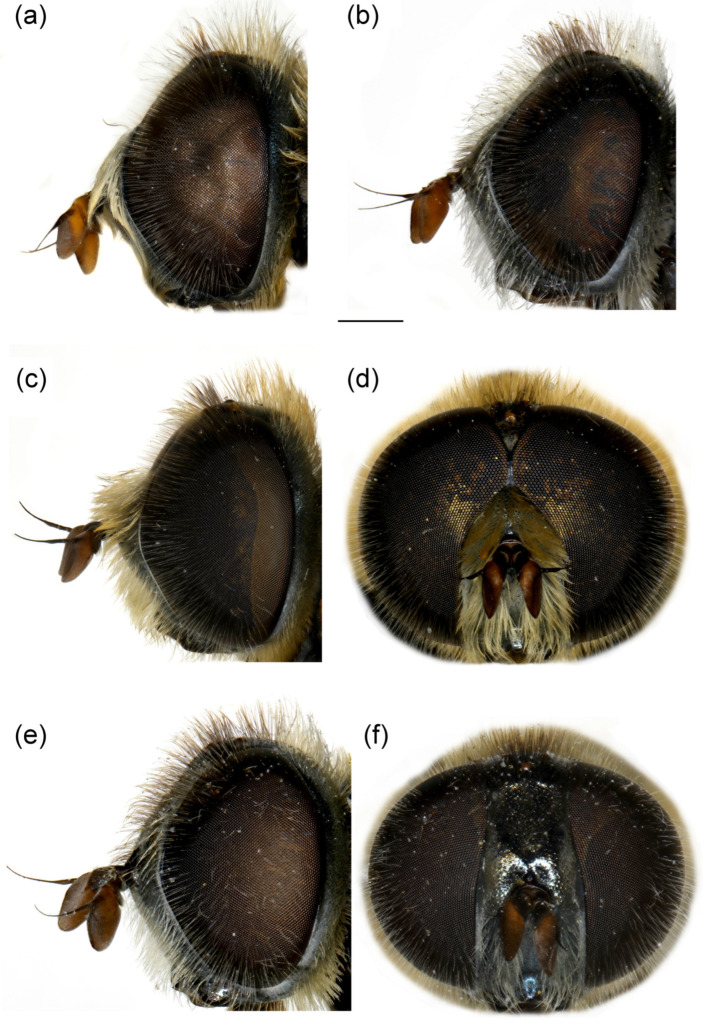
Head of (**a**,**b**) *M. ottomanus*; (**c**–**f**) *M. auriolus* sp. nov. (**a**,**c**,**e**) lateral view; (**b**,**d**,**f**) frontal view. (**a**,**c**,**d**) male; (**b**,**e**,**f**) female. Scale bar 1 mm.

**Figure 11:**
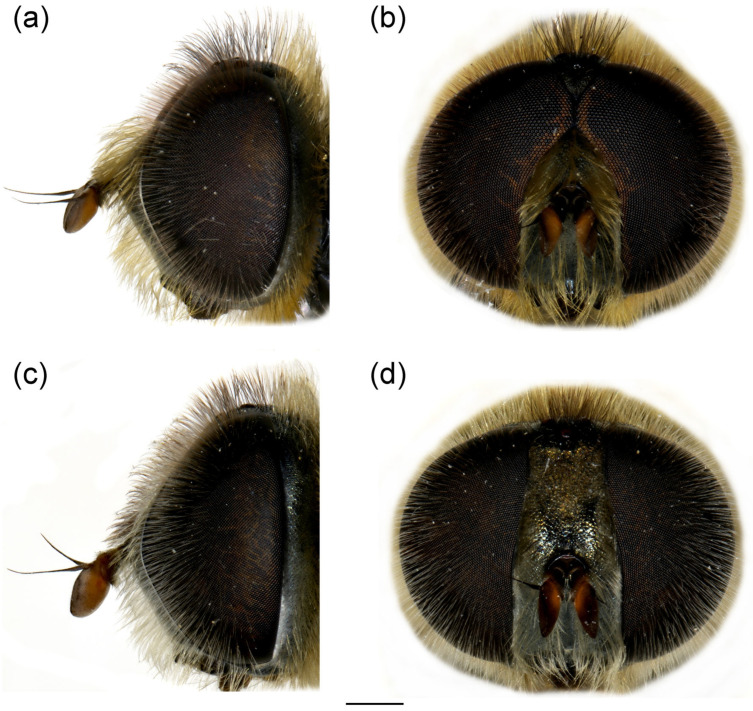
Head of *Merodon paeninsula* sp. nov. (**a**,**c**) lateral view; (**b**,**d**) frontal view. (**a**,**b**) male; (**c**,**d**) female. Scale bar 1 mm.

**Figure 12:**
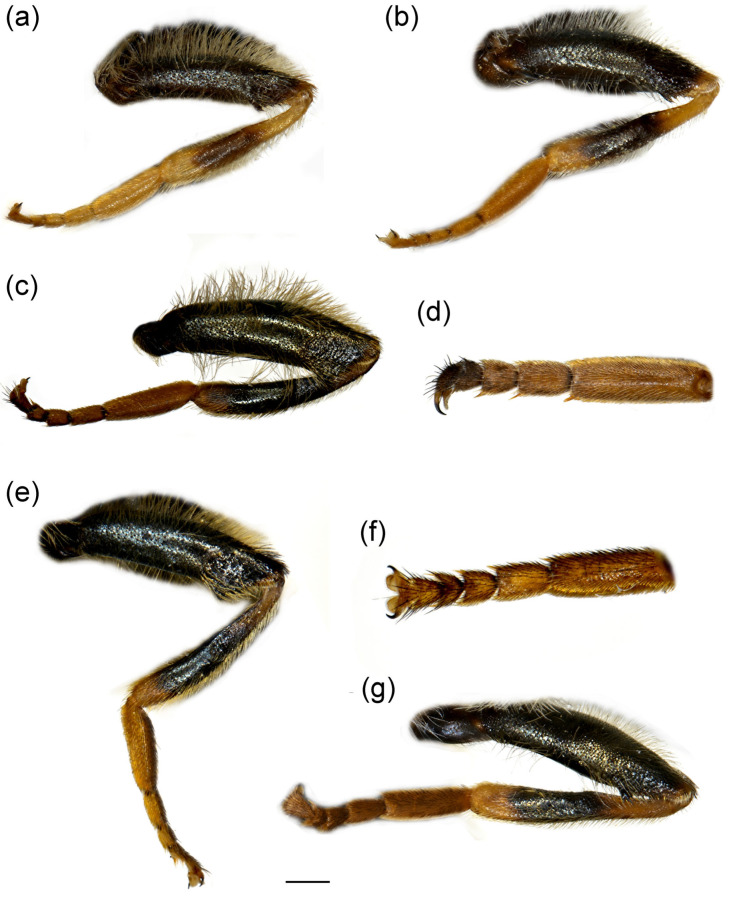
Parts of legs. (**a**) *Merodon ottomanus*, metaleg of male; (**b**) *M. ottomanus*, metaleg of female; (**c**) *M. auriolus* sp. nov., metaleg of male; (**d**) *M. auriolus* sp. nov., metatarsus of male; (**e**) *M. paeninsula* sp. nov., metaleg of male; (**f**) *M. paeninsula* sp. nov., metatarsus of male; (**g**) *M. paeninsula* sp. nov., metaleg of female. (**a**–**c**,**e**,**g**) lateral view; (**d**,**f**) dorsal view. Scale bar (**a**–**c**,**e**,**g**) 1 mm; (**d**,**f**) 0.75 mm.

**Diagnosis**. Species characterised by the large, oval or rounded posterior surstylar lobe of the male genitalia (as in Figure 9a: pl), and the very small anterior surstylar lobe (as in Figure 9a: al). Includes medium-sized species (7–12 mm), with bronze reflections, characterised by the black shiny terga. Body covered with long yellowish to whitish pilosity; pile on frons and face dense and very long (as in Figure 10c and Figure 11a); basoflagellomere elongated, at least partly reddish-yellow ventrally; eye contiguity between 3 and 14 facets long; terga non pollinose or with narrow and weak silvery grey pollinose fasciae, more distinct in females; metafemur ventrally covered with long pile (as in Figure 12c), metabasotarsomere more than 3× longer than wide (as in Figure 12e).

This group has a fragmented range from the Iberian Peninsula through the Peloponnesus (Greece) and Turkey until Iran. Five species are included under the present concept of the group.

**

***Merodon auriolus*** **Vujić, Likov et Radenković sp. nov.**

urn:lsid:zoobank.org:act:197E4699-EE3E-4E6B-8291-3825DE089C4A

**Material examined**. **Holotype**: 1♂, SPAIN, Sierra Nevada, first valley, 37.128217° N 3.445954° W, 20.v.2022, leg. Vujić, FSUNS (FSUNS ID 68467).

**Paratypes**: SPAIN: 3♂♂, Alicante, Font Roja (Alcoy), 10.v.1994, leg. Isidro P.M., [det. as *M. ottomanus* by M.A. Marcos García], CEUA (CEUA00018116-18118); 1♂, Alicante, Agres, Caveta del Voltor, 1200 m a. s. l., 08–23.v.2001, leg. Pérez-Bañón, Marcos-García, Rojo, [det. as *M. ottomanus*, DNA voucher, CIBIO, Ximo Mengual X-27], CEUA (CEUA00002077); 1♀, Alicante, Agres, FoiaAmpla, 23–25.vi.2001, leg. Pérez-Bañón, Marcos-García, Rojo, [det. as *M. ottomanus*, DNA voucher CIBIO, Ximo Mengual X-56], CEUA (CEUA00002071); 4♂♂, Granada, Sierra Nevada, Güejar Sierra, El Dornajo, parking camino a Peña del Perro, 1895 m a. s. l., 24.vi.2021, leg. Nedeljković Z., [det. as *Merodonottomanus* by Z. Nedeljković 2021, on flowers of *Euphorbia nicaeensis* All.], CEUA (CEUA00110748, 110751-110753); 2♂♂, same data as for preceding, leg. Ricarte A., CEUA (CEUA00110767, 110770); 4♀♀, same data as for preceding, CEUA (CEUA00110760, 110761, 110766, 110769); 1♀, same data as for preceding, leg. Aguado Aranda P., CEUA (CEUA00110757); 2♂♂, same data as for preceding, 26.vi.2021, CEUA (CEUA00110749, 110768); 3♀♀, same data as for preceding, leg. Ricarte A., CEUA (CEUA00110754, 110756, 110762); 4♀♀, same data as for preceding, leg. Nedeljković Z., CEUA (CEUA00110755, 110758, 110759, 110763); 1♂, Granada, Sierra Nevada, Güejar Sierra, El Dornajo, Camino a Peña del Perro, 1855 m a. s. l., 21.vi.2021, leg. Ballester Torres I., [det. as *Merodon ottomanus* by Z. Nedeljković 2021], CEUA (CEUA00110750); 1♀, same data as for preceding, leg. Aguado Aranda P., CEUA (CEUA00110764); 1♀, same data as for preceding, leg. Ricarte A., CEUA (CEUA00110765); 2♂♂, Jaén, Sierra de Cazorla, Tornillos de Gualay, 14.vi.1990, leg. Herrera C.M., [det. as *Merodon* aff *ottomanus* by A. Ricarte in 2019], CEUA (CEUA00106927, 106943); 3♂♂, Jaén, P.N. Cazorla, Vadillo Castril, Arroyo Tornillos de Gualay, [37°52′2″ N, 2°55′35″ O], 1575 m a. s. l., 14.vi.2019, leg. Marcos García M.A., [det. as *Merodon ottomanus* by A. Ricarte 2021], CEUA-CIBIO; 1♂, same data as for preceding, leg. Ricarte A., [code INV09537 and det. in 2019], CEUA-CIBIO; 1♂, Jaén, P.N. Cazorla, Vadillo Castril, Barranco de la Cabrilla, turbera, [37°55′48.1″ N, 2°46′57.2″ O], 1639 m a. s. l., 15.vi.2019, leg. Marcos García M.A., [det. as *Merodon ottomanus* by A. Ricarte 2021], CEUA-CIBIO; 1♂, Sierra Nevada, second valley, 37.1027780° N 3.45527799° W, 1434 m a. s. l., 17.vi.2014, leg. Vujić, Radenković, Pérez-Bañón, FSUNS (FSUNS ID 07415, ME336, AU1180); 1♂, Sierra Nevada, first valley, 37.12777799° N 3.44555599° W, 1626 m a. s. l., 17.vi.2014, leg. Vujić, Radenković, Pérez-Bañón, FSUNS (FSUNS ID 07347, ME330, AU1183); 5♀♀, same data as for preceding, FSUNS (FSUNS ID 07322, ME332; FSUNS ID 07314, ME333, AU1181; FSUNS ID 07387, ME334; FSUNS ID 07317, ME335; FSUNS ID 07350, AU1182); 4♂♂, Sierra Nevada, first valley, 37.128217° N 3.445954° W, 20.v.2022, leg. Vujić, FSUNS (FSUNS ID 68468, ‘RU457’; FSUNS ID 68469–68471); 1♀, same data as for preceding, FSUNS (FSUNS ID 68472); 9♂♂, La Corte, 37.961330° N 6.819116° W, 28.iv.2015, leg. Vujić, Obreht, FSUNS (FSUNS ID 09338, ME331, AU1186; FSUNS ID 09339, ME337; FSUNS ID 09332, ME338; FSUNS ID 09335, ME339, AU1184; FSUNS ID 09341; FSUNS ID 09336, ME340; FSUNS ID 09342, AU1177; FSUNS ID 09344, ME341; FSUNS ID 69102, AU1185); 1♀, Andalusia, Siera de Segura, La Hoya, Cambron, 38.2280555° N 2.65305555° W, 1520 m a. s. l., 07.vi.2003, leg. Doczkal, DD coll. (FSUNS ID 02581, DD-05-34); 1♂, Valencia, Utiel, 10.v.1994, 39.5666667° N 1.2° W, leg. Pérez-Bañón C., CEUA-CIBIO (FSUNS ID 02580, ME329); 1♀, same data as for preceding, 09.v.1994, (FSUNS ID 02583); 1♂, Valencia, Utiel, 10.v.1994, leg. C. Pérez-Bañón, [det. as *M. ottomanus* by M.A. Marcos García], CEUA (CEUA00018115); 2♀♀, same data as for preceding, CEUA (CEUA00018123, 18124); 2♀♀, same data as for preceding, 09.v.1994, CEUA (CEUA00018127, 18125); 3♀♀, Valencia, Chelva, 25.iv.–09.v.1994, leg. Pérez-Bañón C., [det. as *M. ottomanus* by M.A. Marcos García], CEUA-CIBIO (CEUA00018120-18122). *All specimens from Alicante and Valencia from the nineties and 2001 were already reported by Marcos-García et al. (2007) as *M. ottomanus*.

**Figure 13:**
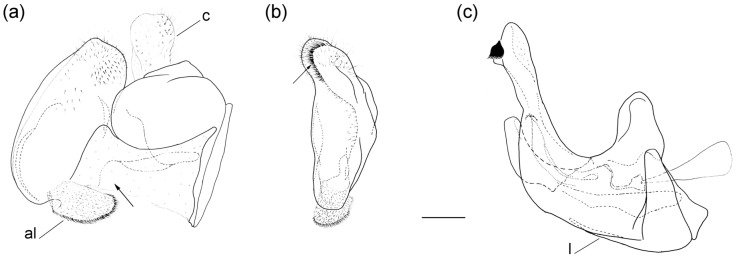
Male genitalia of *Merodon auriolus* sp. nov. (**a**) lateral view of epandrium; (**b**) ventral view of epandrium; (**c**) lateral view of hypandrium. Abbreviations: al—anterior surstylar lobe; c—cercus; l—lingula. A strong marginal spine (**a**) and the black spinae (**b**) are marked with an arrow. Scale bar 0.5 mm.

**Figure 14:**
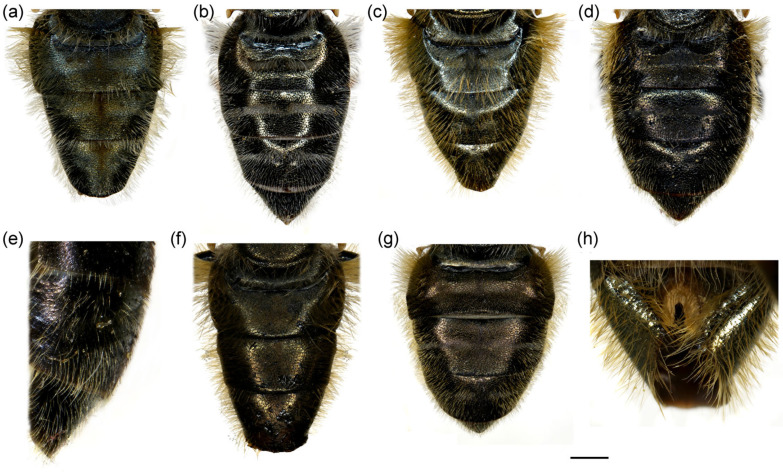
Abdomen of (**a**,**b**) *Merodon ottomanus*; (**c**–**e**,**h**) *M. auriolus* sp. nov.; (**f**,**g**) *M. paeninsula* sp. nov. (**a**,**c**,**f**,**h**) male; (**b**,**d**,**e**,**g**) female. (**a**–**d**) dorsal view; (**e**) lateral view; (**h**) ventral view. Scale bar (**a**–**d**,**f**,**g**) 1 mm; (**e**) 1.5 mm; (**h**) 0.8 mm.

**Diagnosis**. Basoflagellomere reddish-yellow ventrally and dark brown dorsally (Figure 10c); tibiae mostly black, basally and apically brownish red; tarsi reddish-yellow to reddish-brown (Figure 12c); metabasitarsus covered dorsally with light yellow, adpressed pilosity, in some specimens mixed with black ones (Figure 12d); male genitalia: anterior surstylar lobe with a strong marginal spine (Figure 13a: marked with arrow); posterior surstylar lobe large, oval, directed backwards, with black spine innerly (Figure 13b: marked with arrow); female: scutum without pollinose vittae; abdomen rounded (Figure 14d); terga 2–4 without or with indistinct silvery grey pollinose fasciae; tergum 4 postero-medially with black pile.

**Description**

**Male**

**Head**. Basoflagellomere reddish-yellow ventrally and dark brown dorsally (Figure 10c), elongated, about 2× as long as wide, more or less convex dorsally, tapering to the apex; fossette dorso-lateral and large; arista black, thickened at basal third (Figure 10d); face and frons black with grey microtrichia; face covered with long and dense yellow to whitish pilosity; pile on frons dense, long, yellow-reddish; oral margin small, black (Figure 10c), with sparse microtrichia; lunula shining black to brown, bare; eye contiguity about 10–12 facets long; vertex isosceles, dull, black, anterior angle covered with dark grey pollinosity; vertex with grey to yellow-reddish pilosity and black pile around equilateral ocellar triangle; occiput with yellow to reddish pile, covered with a dense, grey microtrichia in lower half; eyes covered with long, dense whitish-grey pile (Figure 10c).

**Figure 15:**
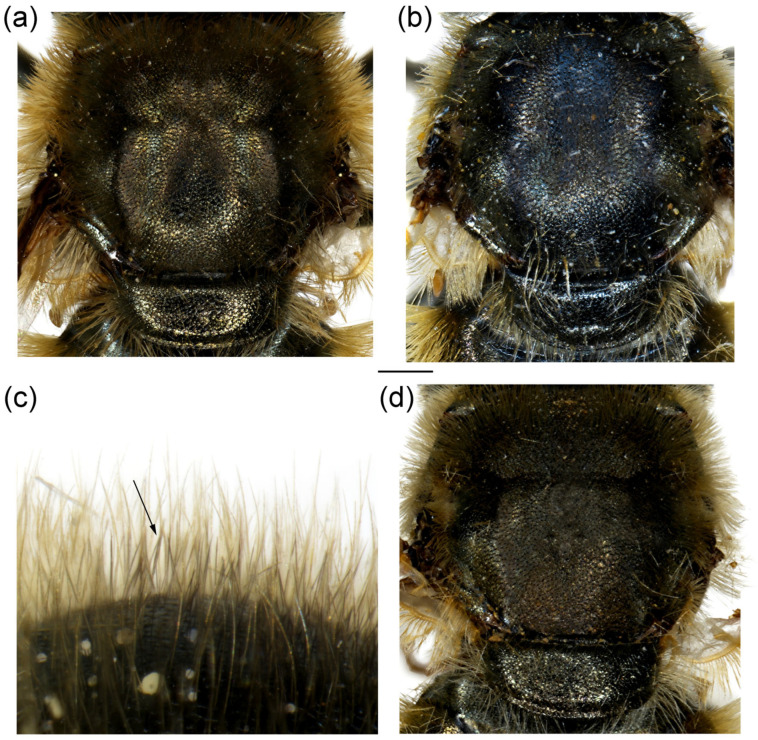
Dorsal view of thorax. (**a**,**b**) *Merodon auriolus* sp. nov.; (**c**,**d**) *M. paeninsula* sp. nov. (**a**,**c**) male; (**b**,**d**) female. The black pile are marked with arrow. Scale bar 1 mm.

**Thorax**. Scutum and scutellum black with bronze to brown lustre, covered with long, dense, erect yellow reddish pile (Figure 15a); scutum dull, without or with indistinct pollinose vittae; posterodorsal part of anterior anepisternum, posterior anepisternum (except anteroventral angle), anterior anepimeron, dorsomedial anepimeron, and posterodorsal and anteroventral parts of katepisternum with long, dense pale yellow pile; wings mostly covered with microtrichia; wing veins brown; calypter yellowish; halter yellow to brown; femora black, covered with long pilosity; tibiae mostly black, yellow to brown-red basally and apically; tarsi yellow-red to reddish-brown; basitarsus of metaleg more than 3× longer than wide, covered dorsally with light yellow adpressed pilosity, in some specimens mixed with black ones (Figure 12d).

**Abdomen**. About 1.2× longer than mesonotum; terga dark brown to black; terga without or with narrow and weak silver/grey pollinose fasciae; pile on terga long, dense, erect, grey-yellow to reddish; sterna dark brown, covered with long whitish-yellow pile; sternum 4 with triangular or oval posterior margin (Figure 14h).

**Male genitalia**. Anterior surstylar lobe small, oval, about 1.5× longer than wide, covered with dense, short pile and with strong marginal spinae (Figure 13a: marked with arrow); posterior surstylar lobe large, oval, directed backwards, with black spinae innerly (Figure 13b: marked with arrow); cercus rectangular (Figure 13a: c); hypandrium sickle-shaped, without lateral projections; lingula large (Figure 13c: l).

**Female**. Similar to male except for typical sexual dimorphism and for the following features: body pilosity mostly whitish; basoflagellomere about 1.8× longer than wide (Figure 10e); frons with pollinose vittae along eye margins variable in shape and size (Figure 10f); abdomen rounded (Figure 14d); terga covered with shorter grey-whitish to yellow pilosity; medial part of terga 2–4 with short black pile; pollinose fasciae on terga 2–4 more distinct than in male.

**Etymology**. The specific epithet is formed from ‘aureus’, the Latin for golden, made of gold, gold coloured, or beautiful, brilliant, excellent, splendid.

**Figure 16:**
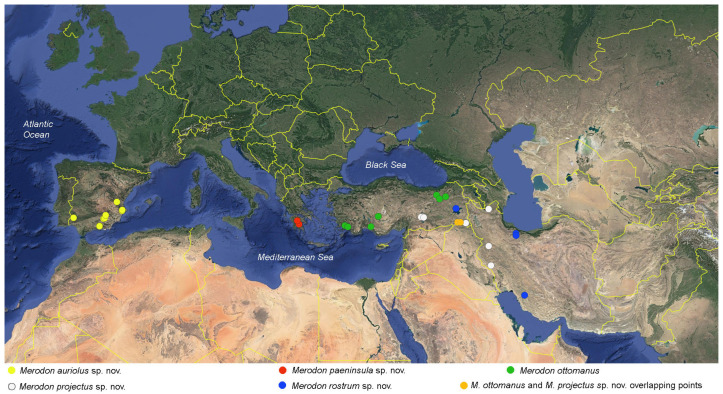
Distribution map of the *Merodon ottomanus* species group.

**Distribution.** Southern parts of the Iberian Peninsula (Figure 16).

**Biological data**. Adults found visiting flowers of *Euphorbia nicaeensis* All.

**

***Merodon ottomanus*** **Hurkmans, 1993**

**Material examined**. **Holotype**: 1♂, TURKEY, Hakkari, Tanin—Tanin pass, 37.4983333° N 42.9783333° E, 2200 m a. s. l., 12.vi.1984, leg. Lucas J.A.W., NBCN.

**Paratypes**: 6♂♂, 1♀, same data as for holotype (1♂ FSUNS ID 02579).

**Additional material**: TURKEY: 1♀, Mugla, 14 km NE from Agla, Lake Kartar, 37.0305556° N 28.7525° E, 1600 m a. s. l., 31.v.2000, leg. Smit J.T. [*Merodon* cf. *minutus* by Hurkmans, 2002], STJ. coll. (FSUNS ID 04062, ME324); 1♂, Antalya, Akseki, Göktepe high plateau, 37.0500° N 31.7333° E, 2100 m a. s. l., A32, 13.vii.1999 [det. as *Merodon ottomanus* by Vujić 2009, published in Vujić et al. (2011)], CEUA-CIBIO; 1♂, Mugla, University campus, 37.1616666° N 28.3725° E, 700 m a. s. l., 17-22.v.2011, leg. Barták Miroslav, Kubik Stepan, BM coll. (FSUNS ID 24758, TS611); 1♀, same data as for preceding, BM coll. (FSUNS ID 24757, DNA ID TS529); 1♀, Hakkari, Suvarihalil Pass, 37.5000° N 43.3333° E, 2100 m a. s. l., 14.vi.1984, leg. Lucas J.A.V., NBCN (FSUNS ID 02582, ME325); 1♂, Bozdag Mountain, Karaomerler, NE Konya, 38.101667° N 32.685° E, 1150 m a. s. l., 23.iv.2001, leg. Lange C., Ziegler J. [*Merodon* aff. *unguicornis* by Romig, 2001], FSUNS (FSUNS ID 09695, ME327); 1♀, same data as for preceding, FSUNS (FSUNS ID 09696, ME326); 1♂, Kop mountain pass [Kop Dağıgeçidi], Bayburt, 40.25° N 40.25° E, 16.vii.1992, NBCN; 1♀, Erzurum, Tepebasi/Askale, 2000 m a. s. l., 08.vi.1996, leg. Hayat R., EMIT (FSUNS ID 62794, ME323); 1♂, 2♀♀, Erzurum, Kosk koyu; 20.vi.1996; leg. Hurkmans W. [‘press. by W. Hurkmans BMNH(E) 1996-180’], BMNH.

**Diagnosis**. Basoflagellomere reddish-yellow, with brownish fossette (Figure 10a); tibiae mostly reddish, dark brown medially; all tarsi reddish-yellow; metabasitarsus covered dorsally with light yellow adpressed pilosity (Figure 12a), while in *M. paeninsula* sp. nov. and usually in *M. auriolus* sp. nov. mixed with black ones (Figure 12f); male genitalia: anterior surstylar lobe with fine marginal spinae (Figure 9a: al); posterior surstylar lobe large, oval, directed backwards, without strong black spinae on the inner side (Figure 9a: pl); female: scutum without pollinose vittae; abdomen slightly elongated (Figure 14b); terga 2–4 with distinct silvery grey pollinose fasciae; tergum 4 postero-medially with black pile.

**Distribution.** Anatolian Peninsula (Figure 16).

**Biological data.** No data.

***Merodon paeninsula* Vujić, Likov et Radenković sp. nov.**

urn:lsid:zoobank.org:act:04AD602D-D1F1-4043-AD44-FA43527FB6AF

**Material examined**. **Holotype**: 1♂, GREECE, Peloponnese, Karyes, 37.303562° N 22.418929° E, 01.v.2022, leg. Vujić, Gorše, FSUNS (FSUNS ID 64964, ME351).

**Paratypes**: GREECE: 2♂♂, same data as for holotype, FSUNS (FSUNS ID 64965, ME352; FSUNS ID 64966, ME353); 1♀, same data as for preceding, FSUNS (FSUNS ID 64967); 3♀♀, Peloponnese, Karyes, 25 km N from Sparta, 37.304145° N 22.421341° E, 905 m a. s. l., 20.v.2016, leg. Vujić, Nedeljković, Ačanski, Likov, Miličić, FSUNS (FSUNS ID 11605, FIN66, ME346; FSUNS ID 11606, TS613, ME346; FSUNS ID 11607, TS615, ME345); 1♀, same data as for preceding, 22.v.2016, FSUNS (CEUA-CIBIO ID 11637, FSUNS ID 11659, TS612, ME343); 1♀, same data as for preceding, 23.v.2016, FSUNS (FSUNS ID 11409, TS614, ME342); 3♀♀, Peloponnese, Karyes, 25 km N from Sparta, 23.v.2014, 37.304160° N 22.42106° E, 933 m a. s. l., leg. Vujić, Ačanski, FSUNS (FSUNS ID 06536, AU1178, ME344; FSUNS ID 06538, ME347; FSUNS ID 06540, AU1179, ME348); 1♀, Mountain Mainalo, Vitina, 37.6666667° N 22.1833333° E, 29.iv.1970, NBCN (FSUNS ID 02584, ME350).

**Diagnosis**. Basoflagellomere reddish-yellow ventrally and dark brown dorsally (Figure 11a); tibiae mostly black, basally and apically brown-red; tarsi reddish-yellow to reddish-brown; metabasitarsus covered dorsally with mixed light yellow and black adpressed pilosity (Figure 12f), in *M. ottomans* metabasitarsi covered only with whitish to light yellow pile; scutum without distinct pollinose vittae, with black pile medially (Figure 15c: marked with arrow), in *M. auriolus* sp. nov. and *M. ottomanus* scutum entirely covered with reddish yellow pilosity; abdomen rounded (Figure 14f); terga 2–4 without or with indistinct silver grey pollinose fasciae; terga 2–4 posterior-medially with black pile.

**Description**

**Male**

**Head**. Basoflagellomere reddish-yellow ventrally and dark brown dorsally (Figure 11a), elongated, about 2× as long as wide, more or less convex dorsally, tapering towards the apex; fossette dorso-lateral and large; arista black, thickened at basal third (Figure 11a); face and frons black with grey microtrichia; face covered with long and dense yellow pilosity; pile on frons dense, long, yellow-reddish; oral margin small, black (Figure 11a), with sparse microtrichia; lunula shining black to brown, bare; eye contiguity about 10 facets long; vertex isosceles, dull, black, anterior angle covered with dark grey pollinosity; vertex covered with black; ocellar triangle equilateral; occiput with yellow to reddish pile, covered with a dense, grey microtrichia in lower half; eye covered with long, dense brown-grey pile (Figure 11a,b).

**Thorax**. Scutum and scutellum black with bronze to brown lustre, covered with long, dense, erect yellowish pile, except black pile on scutum medially (Figure 15c, marked with arrow); scutum dull, without or with indistinct pollinose vittae; posterodorsal part of anterior anepisternum, posterior anepisternum (except anteroventral angle), anterior anepimeron, dorsomedial anepimeron, and posterodorsal and anteroventral parts of katepisternum with long, dense pale yellow pile; wings mostly covered with microtrichia; wing veins brown; calypter yellowish; halter yellow to brown; femora black, covered with long yellowish pilosity; tibiae mostly black, yellow to brownish red basally and apically; tarsi yellowish red to reddish-brown; basitarsus of metaleg more than 3× long as wide, covered with light yellow adpressed pilosity, dorsally mixed with black ones (Figure 12f).

**Abdomen**. About 1.2× longer than mesonotum; terga dark brown to black; terga without silvery grey pollinose fasciae; pile on terga long, dense, erect, greyish yellow; terga 2–4 with some black pile on posterior margin medially (Figure 14f); sterna dark brown, covered with long whitish-yellow pile; sternum 4 with triangular posterior margin.

**Figure 17:**
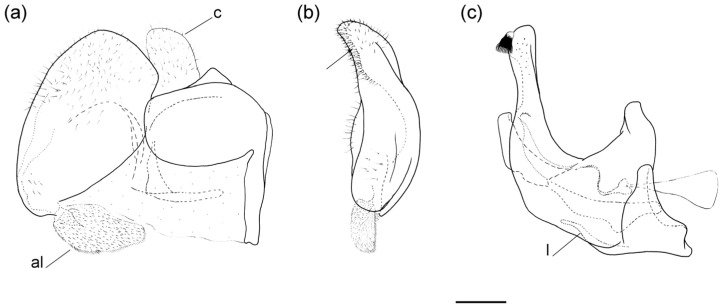
Male genitalia of *Merodon paeninsula* sp. nov. (**a**) lateral view of epandrium; (**b**) ventral view of epandrium; (**c**) lateral view of hypandrium. Abbreviations: al—anterior surstylar lobe; c—cercus; l—lingula. The black spinae are marked with arrow. Scale bar: 0.5 mm.

**Male genitalia**. Anterior surstylar lobe small, oval, about 1.5× longer than wide, covered with dense, short pile and with strong marginal spinae (Figure 17a: al); posterior surstylar lobe large, oval, directed backwards, with black spinae innerly (Figure 17b, marked with arrow); cercus rectangular (Figure 17a: c); hypandrium sickle-shaped, without lateral projections; lingula large (Figure 17c: l).

**Female**. Similar to male except for the typical sexual dimorphism and for the following features: eye pile whitish; body pilosity mostly whitish; basoflagellomere about 1.5× longer than wide (Figure 11c); frons with pollinose vittae along eye margins variable in shape and size (Figure 11d); abdomen rounded (Figure 14g); scutum without black pilosity; terga covered with shorter grey-whitish to yellow pilosity; medial part of terga 2–4 with short black pile; pollinose fasciae on terga 2–4 usually distinct.

**Etymology**. The specific epithet ‘paeninsula’ is given after the type locality of this new species which is a peninsula (Peloponnese).

**Distribution**. Peloponnese (Greece) (Figure 16).

**Biological data.** No data.

***Merodon projectus* Vujić, Likov et Radenković sp. nov.**

urn:lsid:zoobank.org:act:7ED6327E-16D8-4212-94AB-D9325C2A3188

**Material examined**. **Holotype**: 1♂, IRAN, Keleybar forest, 38.851283° N 46.998867° E, leg. Khaghaninia, MMH coll. (FSUNS ID 10297, AU1136).

**Paratypes**: IRAN: 2♂♂, Ilam prov., Dinar Kouh, 32.915° N 47.301° E, 1830 m a. s. l., 12.v.2016, leg. Kafka M., BM coll. (FSUNS ID 69103, TS823; FSUNS ID 69104, TS824); 2♂♂, Kurdistan prov., Paniran, 35.015° N 47.007° E, 1450 m a. s. l., 14.v.2016, leg. Kafka M., BM coll (FSUNS ID 69105, TS832; FSUNS ID 69116).

TURKEY: 1♂, Hakkari, Chilo Daglari, N of Oramar, 37.416629° N 44.039282° E, 1400 m a. s. l., 16.vi.1984, leg. Lucas J.A.W., [Holotype od *Merodon melaleuca* (Hurkmans, unpublished name)], NBCN (FSUNS ID 02585); 1♀, Hakkari, Tanin—Tanin pass, 37.4983333° N 42.9783333° E, 1700 m a. s. l., 12.vi.1984, leg. Lucas J.A.V., [Paratype of *Merodon ottomanus* designated by Hurkmans], NBCN (FSUNS ID 04090); 1♀, Hakkari, Suvarihalil Pass [Suvarihalil Gecidi], 37.5000° N 43.3333° E, 2100 m a. s. l., 14.vi.1984, [Paratype of *Merodon melaleuca* (unpublished name)], NBCN (FSUNS ID 02587); 1♀, Adiyaman, Nemrut Dagi, 38.0° N 38.5833333° E, 1500–2100 m a. s. l., 01.vi.1985, NBCN (FSUNS ID 02588); 2♀♀, Adiyaman, Celikhan, 38.035° N 38.2436111° E, 1450 m a. s. l., 01.vii.1986, leg. Lucas J.A.V., NBCN (FSUNS ID 04220, 04219).

**Figure 18:**
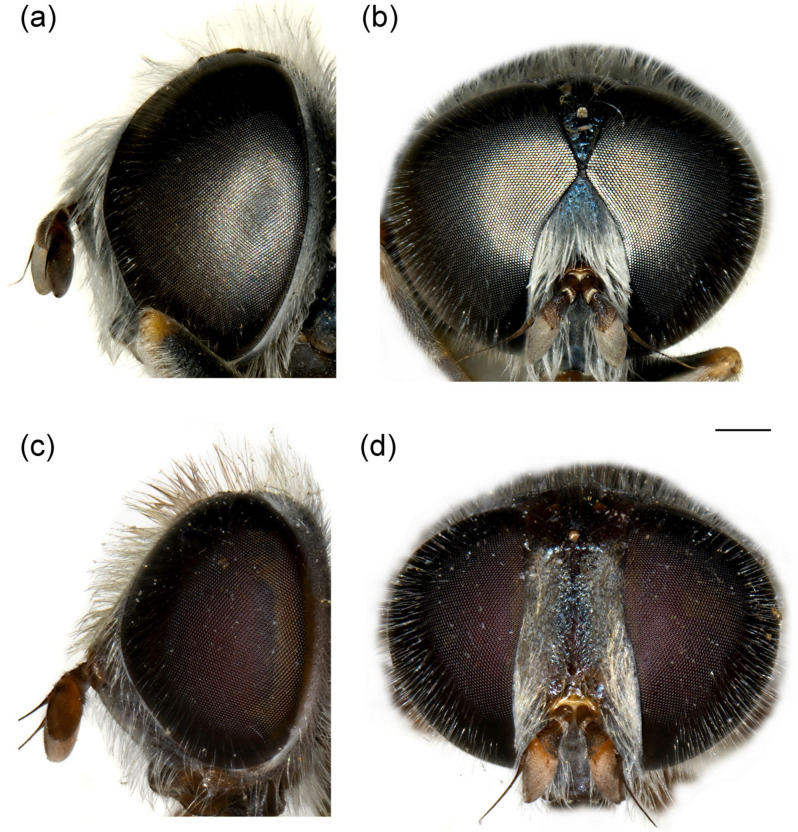
Head of *Merodon projectus* sp. nov. (**a**,**c**) lateral view; (**b**,**d**) frontal view. (**a**,**b**) male; (**c**,**d**) female. Scale bar: 1 mm.

**Figure 19:**
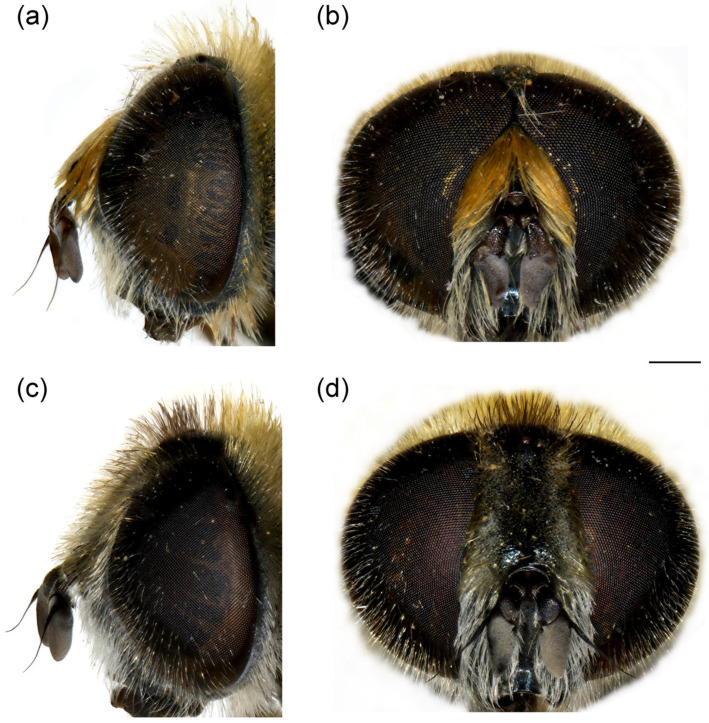
Head of *Merodon rostrum* sp. nov. (**a**,**c**) lateral view; (**b**,**d**) frontal view. (**a**,**b**) male; (**c**,**d**) female. Scale bar: 1 mm.

**Figure 20:**
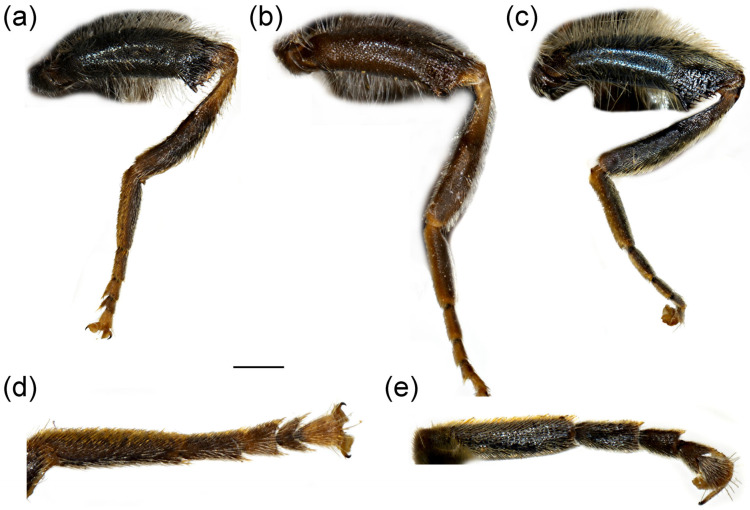
Parts of metalegs. (**a**,**b**), (**d**) *Merodon projectus* sp. nov.; (**c**,**e**) *M. rostrum* sp. nov. (**a**–**c**) lateral view of metaleg; (**d**,**e**) dorsal view of metatarsus. (**a**,**c**–**e**) male; (**b**) female. Scale bar (**a**,**c**,**d**) 1 mm and (**b**,**e**) 2 mm.

**Figure 21:**
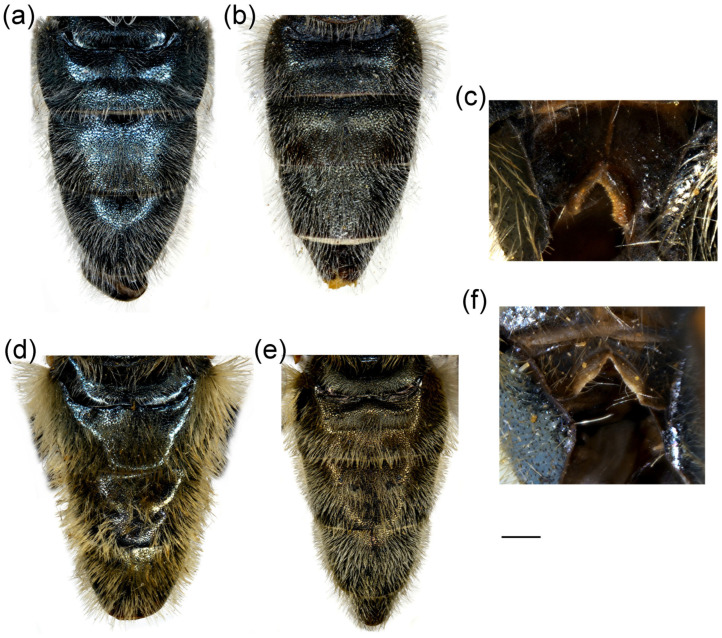
(**a**–**c**) *Merodon projectus* sp. nov.; (**d**–**f**) *M. rostrum* sp. nov. (**a**,**b**,**d**,**e**) dorsal view of abdomen; (**c**,**f**) sternite 4. (**a**,**c**,**d**,**f**) male; (**b**,**e**) female. Scale bar (**a**,**b**,**d**,**e**) 1 mm and (**c**,**f**) 2 mm.

**Figure 22:**
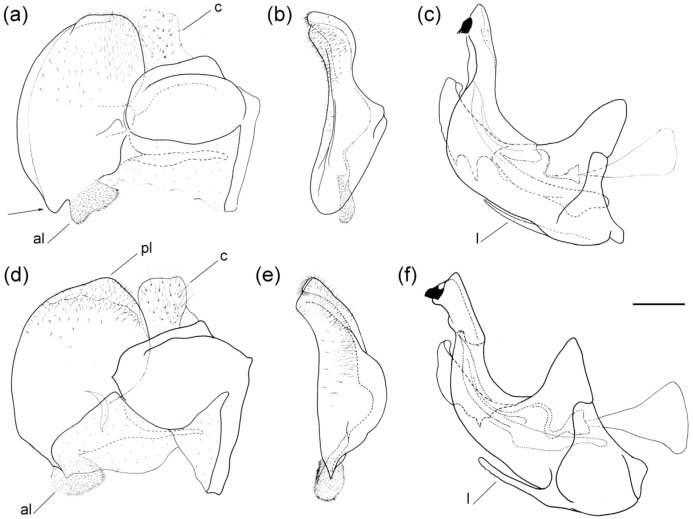
Male genitalia of (**a**–**c**) *Merodon rostrum* sp. nov.; (**d**–**f**) *M. projectus* sp. nov. (**a**,**d**) lateral view of epandrium; (**b**,**e**) ventral view of epandrium; (**c**,**f**) lateral view of hypandrium. Abbreviations: al—anterior surstylar lobe; c—cercus; l—lingula; pl—posterior surstylar lobe. The beak-like protuberance is marked with arrow. Scale bar: 0.5 mm.

**Figure 23:**
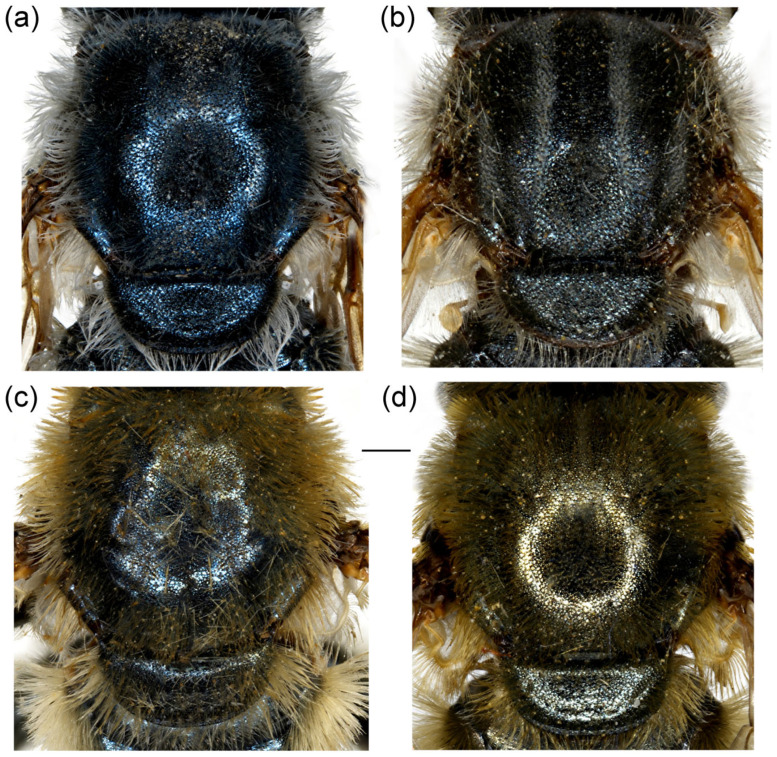
Dorsal view of thorax. (**a**,**b**) *Merodon projectus* sp. nov.; (**c**,**d**) *M. rostrum* sp. nov. (**a**,**c**) male; (**b**,**d**) female. Scale bar: 1 mm.

**Diagnosis**. Basoflagellomere reddish-brown ventrally and dark brown dorsally (Figure 18a,b), contrary to uniformly dark brown in *M. rostrum* (Figure 19 a,c); tibiae mostly black, basally and apically brownish red; tarsi mostly black, at least dorsally; metabasitarsus covered dorsally with light yellow, adpressed pilosity (Figure 20d); male: sternum 4 bare medially, shortly pilose laterally with a pair of wart-like prominences posteriorly (Figure 21c); short eye contiguity about 3–6 facets long; male genitalia: anterior surstylar lobe small, trapezoid (Figure 22d: al); posterior surstylar lobe large, rounded (Figure 22d: pl); female: scutum with distinct pollinose vittae (Figure 23b); abdomen elongated (Figure 21b); terga 2–4 with distinct silvergrey pollinose fasciae; tergum 4 covered with whitish pilosity.

**Description**

**Male**

**Head**. Basoflagellomere brown-reddish ventrally and dark brown dorsally (Figure 18a,b), elongated, about 2× longer than wide, tapering towards the apex; fossette dorsolateral and large; arista dark brown, thickened at basal third (Figure 18b); face and frons black with grey microtrichia, covered with long and dense whitish pilosity; oral margin slightly protruded, black (Figure 18a), with sparse microtrichia; lunula shining black to brown, bare; eye contiguity about 3–6 facets long; vertex isosceles, dull, black, anterior angle covered with dark grey pollinosity; vertex with whitish pilosity and grey pile around equilateral ocellar triangle; occiput with pale yellow pile, covered with dense, grey microtrichia in lower half; eye covered with long, dense whitish-grey pile (Figure 18b).

**Thorax**. Scutum and scutellum black with bronze to bluish lustre, covered with long, dense, erect whitish pile; scutum dull, with more or less distinct pollinose vittae; posterodorsal part of anterior anepisternum, posterior anepisternum (except anteroventral angle), anterior anepimeron, dorsomedial anepimeron, and posterodorsal and anteroventral parts of katepisternum with long, dense whitish pile; wing mostly covered with microtrichia; wing veins light brown; calypter yellowish; halter yellowish; femora black covered with long pilosity; tibiae mostly black, yellow to brown-red basally and apically; tarsi mostly black, at least dorsally (Figure 20a); basitarsus of metaleg more than 3× longer than wide, covered dorsally with whitish, adpressed pilosity (Figure 20d).

**Abdomen**. About 1.2× longer than mesonotum; terga bluish to black; terga without or with narrow weak silvergrey pollinose fasciae; pile on terga long, dense, erect, whitish-grey (Figure 21a); sterna dark brown, covered with long whitish pile; sternum 4 with triangular posterior margin, with a pair of distinct lateral laminate extensions (Figure 21c).

**Male genitalia**. Anterior surstylar lobe small, oval, covered with dense, short pile (Figure 22d: al); posterior surstylar lobe large, oval (Figure 22d: pl); cercus rectangular (Figure 22d: c); hypandrium sickle-shaped, without lateral projections; lingula large (Figure 22f: l). 

**Female**. Similar to male except for the typical sexual dimorphism and for the following features: basoflagellomere about 1.5× longer than wide (Figure 18c); frons with broad pollinose vittae along eye margins (Figure 18d); scutum with four distinct pollinose vittae (Figure 23b); abdomen elongated (Figure 21b); terga covered with whitish-grey to whitish pilosity, shorter than in male; pollinose fasciae on terga 2–4 usually distinct.

**Etymology**. The specific epithet is derived from the Latin ‘projectus’ meaning ‘jutting out, projecting’ and refers to the shape of the male’s posterior margin of sternum 4 bearing two small laminate extensions.

**Distribution**. Southeastern Turkey and Western Iran (Figure 16).

***Merodon rostrum*** **Vujić, Likov et Radenković sp. nov.**

urn:lsid:zoobank.org:act:4B6DC724-3C1F-4D01-B235-02807EFF07F5

**Material examined**. **Holotype**: 1♂, TURKEY, Gevas, lake Van, 38.916780° N 42.772134° E, 29.vi.1993, leg. Halada M., NBCN (FSUNS ID 04099).

**Paratypes**: IRAN: 1♂, Chiraz-Kazeround, 29.6183333° N 51.6583333° E, 2200 m a. s. l., 14.v.1937, leg. Brandl, NBCN (FSUNS ID 02586; LML-05-19), [Holotype of *Merodon citrinus* (Hurkmans, unpublished name) (red label); paratype of *Lampetia*? (unreadable yellow label); *Lampetia? by* P.H. van Doesburg (unreadable label)]; 1♀, Khoznan, 36.122222° N 50.5575° E, 1670 m a. s. l., 05.vi.2014, leg. Sev. and Val. Korneyev, SIZK (FSUNS ID 25114, TS808); 2♂♂, Ghazvin province, Juladak, Moalem kelayeh, Almot, 36.351944444° N 50.53833333° E, 2300–2500 m a. s. l., 30.v.–09.vi.2007, leg. Gharali Babak, FSUNS (FSUNS ID 24866, 24867).

TURKEY: 1♂, Gevas, lake Van, 38.916780° N 42.772134° E, 29.vi.1993, leg. Halada M., NBCN (FSUNS ID 04098); 1♂, same data as for preceding, leg. Denes K., NBCN (FSUNS ID 04097).

**Diagnosis**. Basoflagellomere long, about 2× longer than wide, dark brown (Figure 19a,b), while is reddish-brown ventrally and dark brown dorsally in *M. projectus* (Figure 18a,c); tibiae mostly black, basally and apically brown-red; tarsi mostly black, at least dorsally; metabasitarsus covered dorsally with light yellow, adpressed pilosity (Figure 20e); male genitalia: anterior surstylar lobe small, oval (Figure 22a: al); posterior surstylar lobe broad, rounded (Figure 22a: pl); female: scutum without distinct pollinose vittae (Figure 23c); abdomen elongated (Figure 21d); terga 2–4 with distinct silvery grey pollinose fasciae; tergum 4 covered with whitish pilosity.

**Description**

**Male**

**Head**. Basoflagellomere dark brown (Figure 19a,b), elongated, about 2× longer than wide, more or less convex dorsally, tapering towards the apex; fossette dorsolateral and large; arista dark brown, thickened at basal third (Figure 19a); face and frons black with grey microtrichia; face covered with long and dense whitish pilosity and frons with yellow pile; oral margin slightly protruded, black (Figure 19a), with sparse microtrichia; lunula shining black to brown, bare; eye contiguity about 10 facets long; vertical triangle isosceles, dull, black, anterior angle covered with dark grey pollinosity; vertex with yellowish pilosity; ocellar triangle equilateral; occiput with yellow pile, covered with dense, grey microtrichia in lower half; eye covered with long, dense whitish-grey pile (Figure 19a,b).

**Thorax**. Scutum and scutellum black with bronze to brown lustre, covered with long, dense, erect yellow pile; scutum dull, without pollinose vittae; posterodorsal part of anterior anepisternum, posterior anepisternum (except anteroventral angle), anterior anepimeron, dorsomedial anepimeron, and posterodorsal and anteroventral parts of katepisternum with long, dense pale-yellow to whitish pile; wings mostly covered with microtrichia; wing veins light brown; calypter yellowish; halter yellowish; femora black, covered with long pilosity; tibiae mostly black, yellow to brown-red basally and apically; tarsi mostly black, at least dorsally; basitarsus of metaleg more than 3× longer than wide, covered dorsally with whitish-yellow adpressed pilosity (Figure 20e).

**Abdomen**. About 1.2× longer than mesonotum; terga brown to black; terga without or with narrow and weak silvery grey pollinose fasciae; pile on terga long, dense, erect, yellowish-white; sterna dark brown, covered with long whitish pile; sternum 4 with triangular posterior margin, without distinct lateral laminate extensions (Figure 21d).

**Male genitalia**. Anterior surstylar lobe quadratic, about 1.5× longer than wide, covered with dense, short pile (Figure 22a: al); posterior surstylar lobe more rounded, directed upwards, with distinct beak-like protuberance on ventral margin (Figure 22a: marked with arrow); cercus rectangular (Figure 22a: c); hypandrium sickle-shaped, without lateral projections; lingula large (Figure 22c: l).

**Female**. Similar to male except for the typical sexual dimorphism and for the following features: frons with broad pollinose vittae along eye margins, at the level of ocellar triangle with black pilosity (Figure 19d); abdomen elongated (Figure 21e); terga covered with yellowish to whitish pilosity, shorter than in male; pollinose fasciae on terga 2–4 distinct.

**Etymology**. The noun ‘rostrum’ is given after the character on surstylus male genitalia which is in the shape of a beak (rostrum).

**Distribution**. Iran and eastern Turkey (Figure 16).

**Key to the species of *Merodon ottomanus* group**

1. Metabasitarsus covered with dense black spinae (as in Figure 12f) .........................................................................................................................................................**2**
- Metabasitarsus covered only with pale yellow pile (as in Figure 20d) .........................................................................................................................................................**3**
2. Scutum mostly shiny; distribution: Iberian Peninsula; molecular data (Figure 25)..............................***Merodon auriolus*** Vujić, Likov et Radenković sp. nov. (in part)
- Scutum dull, except shiny lateral margins; distribution: Peloponnese (Greece); molecular data (Figure 25) ................... ***Merodon paeninsula*** Vujić, Likov et Radenković sp. nov.
3. Males .................................................................................................. **4**
- Females .................................................................................................. **7**
4. Sternum 4 bare medially, shortly pilose laterally with a pair of wart-like prominences posteriorly (Figure 21c); male genitalia: anterior surstylar lobe very small and rounded (Figure 22d: al); distribution: Iran and Turkey ... ***Merodon projectus*** Vujić, Likov et Radenković sp. nov.
- Sternum 4 mostly covered with long pile and without wart-like prominences posteriorly (as in Figure 21f); male genitalia: anterior surstylar lobe larger and with a different shape (more quadratic or oval) (Figure 22a: al) .................................................................................................................... **5**
5. Male genitalia: posterior surstylar lobe more rounded, directed upwards, with distinct beak-like protuberance on ventral margin (Figure 22a: indicated with an arrow) ............................................. ***Merodon rostrum*** Vujić, Likov et Radenković sp. nov.
- Male genitalia: posterior surstylar lobe more oval, elongated, directed backwards (as in Figure 13a), without a distinct beak-like protuberance .......................................................................................................................................................... **6**
6. Male genitalia: posterior surstylar lobe with black spinae on the inner side (Figure 13b: indicated with an arrow); anterior surstylar lobe with strong marginal spinae (Figure 13a: al); distribution: Iberian Peninsula ........................................ *Merodon auriolus* Vujić, Likov et Radenković sp. nov. (in part)
- Male genitalia: posterior surstylar lobe without black spinae on the inner side (Figure 9b), anterior surstylar lobe with weak marginal spinae (Figure 9a: al); distribution: Anatolian Peninsula ....................................................... ***Merodon ottomanus***
7. Metabasitarsus yellow, reddish or brown dorsally, opposite to black/dark brown colour of medium part of metatibia (as in Figure 12c,d); abdomen rounded (as in Figure 14c); basoflagellomere usually yellow to red with brown dorsal margin (as in Figure 10c); tergum 4 posteromedially covered with black pile ......................................................................................................................................................... **8**
- Metabasitarsus black to dark brown dorsally, of the same colour as the medium part of metatibia (as in Figure 20e); abdomen elongated (as in Figure 21a); basoflagellomere usually brown or dark brown (as in Figure 19b); tergum 4 completely covered with whitish-yellow pilosity .............................................................................................................. **9**
8. Basoflagellomere yellow to red ventrally and brown dorsally (Figure 10e,f); tarsi reddish-yellow; distribution: Iberian Peninsula ........................ ***Merodon auriolus*** Vujić, Likov et Radenković sp. nov.
- Basoflagellomere yellow with a brown fossette (as in Figure 10a); tarsi bright yellow; distribution: Anatolian Peninsula .................................... ***Merodon ottomanus***
9. Basoflagellomere shorter and smaller, reddish-brown ventrally and dark brown dorsally (Figure 18c,d); mesoscutum usually with 5 distinct pollinose longitudinal vittae (Figure 23b); distribution: Iran and Turkey ............................................................................... ***Merodon projectus*** Vujić, Likov et Radenković sp. nov.
- Basoflagellomere longer and larger, uniformly dark brown (Figure 19c,d); mesoscutum without or with less distinct pollinose longitudinal vittae (Figure 23d) .............................................................................................................................***Merodon rostrum*** Vujić, Likov et Radenković sp. nov.

### 3.2. Geometric Morphometrics

**Figure 24:**
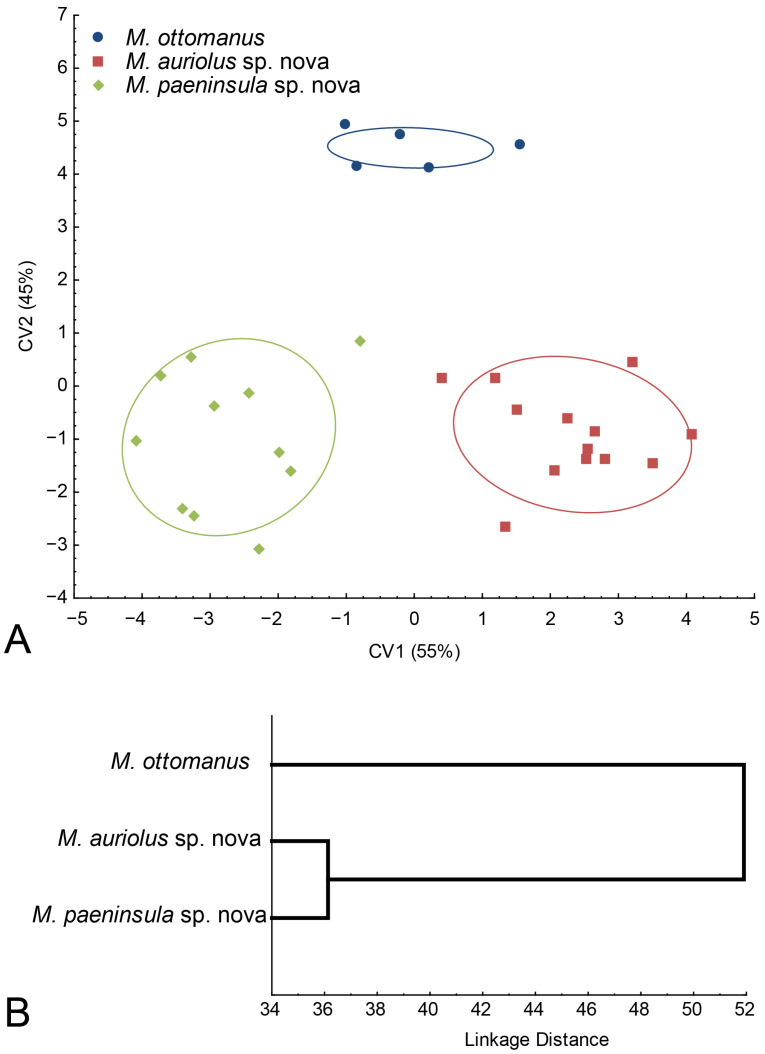
Wing shape differences among *Merodon auriolus* sp. nov., *M. paeninsula* sp. nov. and *M. ottomanus*. (**A**) Scatter plot of individual scores of CV1 and CV2; (**B**) UPGMA phenogram constructed using squared Mahalanobis distances of wing shape.

Since the morphological differences among *M. auriolus* sp. nov., *M. paeninsula* sp. nov., and *M. ottomanus* are discrete, we employed geometric morphometric analysis of wing shape to support these species concepts. A discriminant analysis showed that all three species differed highly significantly in wing shape (*p* < 0.01). In addition, classification success was excellent, with all specimens correctly classified to a priori defined groups. Next, the CVA conducted on the shape variables produced two highly significant canonical axes (CV1: Wilks’ λ = 0.0256, χ^2^ =76.946, *p*< 0.01; CV2: Wilks’ λ = 0,1755, χ^2^ = 36.547, *p* < 0.01). All three species were clearly separated in the space defined by CV1 and CV2 (Figure 24A). CV1, with 55% of total wing shape variations, separated *M. auriolus* sp. nov. from *M. paeninsula* sp. nov., whereas CV2, with 45% of total shape variation, separated M. ottomanus from the other two species (Figure 24A).

A UPGMA phenogram based on the squared Mahalanobis distances showed that *M. auriolus* sp. nov. and *M. paeninsula* sp. nov. had the most similar wing shape, whereas *M. ottomanus* had the most different wing shape (Figure 24B).

### 3.3. Molecular Analysis

**Figure 25:**
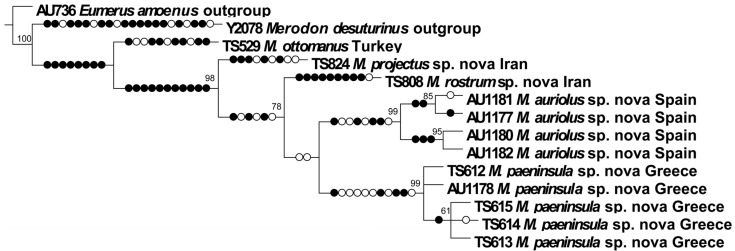
Maximum Parsimony tree based on 5′-COI gene fragment (filled circles stand for unique changes; open circles stand for non-unique changes; bootstrap values ≥ 50 are presented near nodes). Strict consensus tree of two equally parsimonious trees, L = 180, Ci = 77, Ri = 75.

We have analysed molecular data as an additional character to support morphological species concepts of the *M. ottomanus* group. In total, 14 analysed specimens (12 specimens belonging to the examined species group and two outgroups) comprised 602 nucleotide characters of the COI gene with 68 parsimony-informative sites for the total matrix (after aligning and pruning the sequence data). The constructed strict consensus Maximum Parsimony tree (Figure 25) separated all the previously morphologically defined species of the *M. ottomanus* group. The newly described species of the analysed group were clearly separated from each other with high bootstrap supports: *M. projectus* sp. nov. (98), *M. rostrum* sp. nov. (78), *M. auriolus* sp. nov. (99), and *M. paeninsula* sp. nov. (99). Of special importance was the genetic difference between *M. auriolus* sp. nov. and *M. paeninsula* sp. nov., because their morphological separation is difficult. The specimens of these two species were clearly resolved into separate clades, as well as from the other analysed species of the group.

## 4. Discussion

### 4.1. Integrative Taxonomy

Integrative taxonomy combines multiple lines of evidence to define, identify, and classify organisms, including species. In the taxonomy of hoverflies, an integrative approach is advantageous due to the complexity and diversity of this group. Hoverflies are an ecologically significant group of insects, known for their role in pollination and for the diverse functions of their larvae, such as preying on pest insects [45]. Their taxonomy has traditionally been based on morphological characteristics. However, DNA barcoding and other molecular analyses, wing shape analysis, and ecological and geographical data have become indispensable tools for delineating hoverfly species. Integrative taxonomy brings a more robust and nuanced understanding of hoverfly diversity, which is crucial not only due to their role as pollinators but also because of their contribution to the biodiversity and functioning of ecosystems.

This approach was proven invaluable in the study of *Merodon caudatus* and *M. ottomanus*, where an extensive morphological analysis of the existing material, together with DNA barcoding, geometric morphometrics of wing shape, and a comprehensive examination of their distribution patterns, reveals that single species are, in fact, species groups. A detailed morphological examination of specimens labelled as *M. caudatus* and *M. ottomanus* in different entomological collections resulted in the revision of these taxa, including the establishment of two new species groups within the *avidus-nigritarsis* lineage, and the description of five new species. Under the here-presented concept, the *M. caudatus* group consists of two species, *M. caudatus* and *M. crispotarsus* sp. nov. Both species have very distinctive and unique morphological characters to connect them in one monophyletic group, such as the structure of legs and the shape of male genitalia. They clearly differ in the characteristics of legs, male genitalia, and distribution. Unfortunately, all available material is very old, and it was impossible to obtain genetic information, but the morphological characters are very clear and outstanding.

The *Merodon ottomanus* group consists of five species, i.e., *M. projectus* sp. nov., *M. rostrum* sp. nov., *M. auriolus* sp. nov., *M. paeninsula* sp. nov., and *M. ottomanus*. The latter three species showed only subtle morphological differences; however, the geometric morphometric analysis of wing shape confirmed that they were clearly separated. A scatterplot of canonical variates offers a visual method to understand the relationships and distinctions among examined groups. In previous *Merodon* research, the position of individuals within the CVA space differs across the *Merodon* groups and complexes, ranging from distinct separations to slight overlaps, although they are all statistically different in wing shape. Specifically, within the *Merodon avidus* complex [8,15], an overlap indicates the wing shape similarities among the species under study, similar to the slight overlap seen in *Merodon natans* [4], whereas for the *Merodon chalybeus* complex, the spatial arrangement of individuals is clear [25]. Regarding species from the *ottomanus* group, despite the limited number of samples available for analysis, their clear positioning in the CVA space and the high classification accuracy are additional indicators that these species have different wing shapes. Based on the analysed sample, the most similar wings are found in *M. auriolus* sp. nov. and *M. paeninsula* sp. nov., which is in accordance with their position on the Maximum Parsimony tree based on 5′-COI gene fragments.

Additionally, the molecular data further supported the concepts of all new species of the *M. ottomanus* group. Therefore, an integrative taxonomy approach employing multiple data sources was proven to be useful in species delimitation, as was the case in many previous studies concerning *Merodon* (e.g., [4,19,20,27,46]).

### 4.2. Distribution and Diversity

The *Merodon caudatus* species group consists of two species, i.e., *M. caudatus* in the Anatolian Peninsula and *M. crispotarsus* sp. nov., distributed in the western parts of the Levant region. The *M. ottomanus* species group has a larger and more fragmented distributional range. The western part of the range includes many localities of the Iberian Peninsula with only one species from the group present, i.e., *M. auriolus* sp. nov. Another endemic is *M. paeninsula* sp. nov., recorded only in a few localities of the Peloponnese, Greece. The other three species of the group are found throughout the Anatolian Peninsula, i.e., *M. ottomanus*, *M. projectus* sp. nov., and *M. rostrum* sp. nov., with the latter two species spreading further SE, to Iran. The distribution of the newly described *M. projectus* sp. nov. partially overlaps with *M. ottomanus*, with both species recorded in two localities of Hakkari, SE province of Turkey. However, the two species are clearly separated by both molecular data and morphological characters.

The main part of the distributional range of the *Merodon ottomanus* and *M. caudatus* species groups is within the Mediterranean region, where the highest species diversity has been previously recorded within the genus (e.g., [4,16,17,21,47,48,49,50]). The high diversity and number of endemic species havebeen related to the intense orogenic activity, favouring isolation and allopatric speciation among populations, as in the eastern Mediterranean Basin [51]. Most species dealt with in the present study are from the biologically diverse Anatolian region, characterised by a rich geological history, comprising an extensive system of high mountain chains and closed basins, thus providing a wide range of habitats. Throughout history, different parts of this topographically complex area, connecting diverse geographic regions of Asia and Europe, have served not only as natural barriers but also as highly important refugia and corridors providing passages for species spreading [52,53]. Particularly, the Anatolian [51,53] and Iberian [30] peninsulas have been considered centres of diversity and endemism of *Merodon* within the Palaearctic Region. In addition, a number of species havebeen recorded in the broader region of the Middle East in the last decade (e.g., [7,16,52,54]).

Three newly described species in the present study, namely *Merodon auriolus* sp. nov. and *M. paeninsula* sp. nov. (from *M. ottomanus* species group), and *M. crispotarsus* sp. nov. (*M. caudatus* group), have been recorded within the Mediterranean Basin biodiversity hotspot, part of the highly diverse but little-known invertebrate fauna [55]. The geographical distributions of *Merodon* are considered to be linked to thepresence of geophyte-rich flora [7,51,52]. Phytophagous larvae of *Merodon* feed on underground storage organs such as bulbs and corms of the families Asparagaceae, Amaryllidaceae, Iridaceae, and Liliaceae (e.g., [12,13]). The highest geophyte diversity concentrates in the typically Mediterranean-climate areas, where hot dry summers alternate with cool moist winters; the Mediterranean Basin is one of the world’s richest geophyte areas [56]. Moreover, the existence of favourable habitats, various topography and geological substrate, supports the high number of endemic geophytes in areas such as the mountainous southern Balkan Peninsula, including the Peloponnesus [57]. Changes in ecological factors within short distances, due to geomorphic and altitudinal variations, and mountain ranges constituting effective barriers, encourage high species diversity. Anatolia is positioned at the junction of major phytogeographical regions and has historically served as a passage between the continents, resulting in the differentiation and dispersal of a variety of species [58]. Turkey has the richest flora in the temperate zone, with about a third of all plant species being endemic and is among the world’s most biodiverse countries in terms of bulbous monocots [56,58].

Two species newly described in the present study, namely *Merodon projectus* sp. nov. and *M. rostrum* sp. nov., have been recorded within the Irano-Anatolian biodiversity hotspot, which is not well studied in terms of its invertebrate fauna [55]. As noted in a recent publication [6] describing new species of *avidus-nigritarsis* lineage from Iran, a systematic study of the hoverfly fauna in Iran has not been conducted so far;therefore, it is expected that the diversity of hoverflies, including *Merodon*, could be higher in this area. The mountains and basins of the Irano-Anatolian hotspot contain many centres of local endemism, forming a natural barrier between the dry plateaus of Western Asia and the Mediterranean Basin. It hosts high floral diversity with many single-locality endemic plants, most of them threatened [55]. Climatological differences, vegetation history, and geographical isolation make Iran a global centre of plant diversity and an endemic centre of the Irano-Turanian region [59].

Up to date, there is no data on the larval hostplants, immature stages, or breeding and oviposition sites for species in the *Merodon ottomanus* and *M. caudatus* groups. Information on *Merodon* developmental stages is generally very scarce (e.g., [28]). Considering the *avidus-nigritarsis* lineage, the description of preimaginal stages hasbeen published for only two species so far, i.e., *M. opacus* Vujić, Likov et Radenković, suspected to feed on bulbs of *Gagea* Salisb.or *Fritillaria* Tourn. ex L.(Liliaceae) [17], and *M. avidus* (Rossi), associated with bulbs of *Ornithogalum* L. (Asparagaceae) [60,61]. There is a need for further research regarding these plant-insect interactions, in order to understand the relations of *Merodon* species and geophytes as their larval hosts. This is particularly important in terms of potential habitat loss and other risks to host-plant availability. Primary threats to the Mediterranean Basin hotspot’s biodiversity include forest fires, pollution, agricultural intensification and land abandonment, transport infrastructure and residential development due to population migration and the development of the tourist industry [55]. For example, the unique flora and the diverse habitats of Turkey have declined rapidly over the last decades, with the main threats including urban and industrial development, inadequate forest management, overgrazing, extension of intensive agriculture, tourism, and recreational activities [56]. The greatest threat arises from the destruction of habitats, while bulbous plants are additionally affected by commercial collection for the horticultural trade [58]. Similar situations occur for other areas in question, i.e., Iran, where monocots are heavily affected by anthropogenic pressure because of their high ornamental value, and threatened by uncontrolled harvesting [59]. Many threats have led to loss of habitat in the Irano-Anatolian biodiversity hotspot, including increased deforestation, overgrazing, agricultural expansion, mining, and military operations. These impacts resulted in the reduction of forests, wetlands, and steppes; still, alpine meadows covering higher mountains remain largely intact [55].

The five newly described *Merodon* species in the present study add to the current knowledge on the diversity of the genus, and it is to be expected that research in the future will further increase the number of described species, supporting the status of *Merodon* as one of the hoverflies’ most species-rich genera (e.g., [6,7]. The results of the present study contribute to understanding its distribution and confirm previous conclusions highlighting the significance of the Mediterranean region as a hotspot for hoverflies (e.g., [16]), and the importance of underexplored regions, such as Iran, in hosting a high diversity of *Merodon* species [6,7,17,54]. Additionally, these results call attention to the relevance of status reassessment of threatened species in the light of new findings. According to the latest European regional assessment of the IUCN Red List, the species *Merodon ottomanus* has been listed as Vulnerable (VU) within the geographic range of Spain and Greece [62]. However, according to the data revealed in the present study, these are in fact the two species of the *M. ottomanus* group in Europe, namely *M. auriolus* sp. nov. in Spain and *M. paeninsula* sp. nov. in Greece. Thus, updating the information on taxonomy and distribution is crucial for the future reassessments that are vital for determining trends and conservation actions.

## Data Availability

The original contributions presented in this study are included in the article/Supplementary Material. Further inquiries can be directed to the corresponding author.

## Supplementary Materials

The following supporting information can be downloaded at: https://www.mdpi.com/article/10.3390/insects16101009/s1, Table S1. List of molecularly analysed specimens with GenBank accession numbers (in boldface: newly generated sequences within this study).
